# Analysis of Proteome Profile in Germinating Soybean Seed, and Its Comparison with Rice Showing the Styles of Reserves Mobilization in Different Crops

**DOI:** 10.1371/journal.pone.0056947

**Published:** 2013-02-27

**Authors:** Chao Han, Xiaojian Yin, Dongli He, Pingfang Yang

**Affiliations:** 1 Key Laboratory of Plant Germplasm Enhancement and Speciality Agriculture, Wuhan Botanical Garden, Chinese Academy of Sciences, Wuhan, China; 2 Graduate University of Chinese Academy of Sciences, Beijing, China; University of Delhi South Campus, India

## Abstract

**Background:**

Seed germination is a complex physiological process during which mobilization of nutrient reserves happens. In different crops, this event might be mediated by different regulatory and metabolic pathways. Proteome profiling has been proved to be an efficient way that can help us to construct these pathways. However, no such studies have been performed in soybean germinating seeds up to date.

**Results:**

Proteome profiling was conducted through one-dimensional gel electrophoresis followed by liquid chromatography and tandem mass spectrometry strategy in the germinating seeds of soybean (*glycine max*). Comprehensive comparisons were also carried out between rice and soybean germinating seeds. 764 proteins belonging to 14 functional groups were identified and metabolism related proteins were the largest group. Deep analyses of the proteins and pathways showed that lipids were degraded through lipoxygenase dependent pathway and proteins were degraded through both protease and 26S proteosome system, and the lipoxygenase could also help to remove the reactive oxygen species during the rapid mobilization of reserves of soybean germinating seeds. The differences between rice and soybean germinating seeds proteome profiles indicate that each crop species has distinct mechanism for reserves mobilization during germination. Different reserves could be converted into starches before they are totally utilized during the germination in different crops seeds.

**Conclusions:**

This study is the first comprehensive analysis of proteome profile in germinating soybean seeds to date. The data presented in this paper will improve our understanding of the physiological and biochemical status in the imbibed soybean seeds just prior to germination. Comparison of the protein profile with that of germinating rice seeds gives us new insights on mobilization of nutrient reserves during the germination of crops seeds.

## Introduction

Plant seeds, especially crop seeds, accumulate abundant reserves such as carbohydrates, oils and proteins during the maturation [1], which makes them the staple food for the world population. These reserves are also important for seed germination and seedling establishment. The composition of reserves differs dramatically among different plant species. In rice seed, starch accounts for about 85% of the total dry mass [2]. Whereas, *Brassica napus* seed contains about 40% oil and 15% protein, and soybean seed contains about 40% protein and 20% oil [3]. Different reserves are mobilized through different metabolic pathways during germination. The same reserve may be also subject to different degradation pathways in different crops. For example, oils are degraded through classical lipoxygenase (LOX)-independent pathway in rape and corn seeds [4], but through LOX-dependent pathway in cucumber [4], [5].

Germination begins with the water uptake of dry seed, and ends with the emergence of the radicle [6]. Generally, it can be divided into three phases based on the style of water uptake. Phase I is a rapid water uptake phase in which DNA damage repairing [7], [8] and resuming of glycolytic and oxidative pentose phosphate pathways occur [9]. Phase II is a plateau phase in which mitochondria synthesis[9] and translation of storage mRNA occurred [10]. Phase II is also regarded as a metabolism active phase during which reserves mobilization is initiated. Phase III is the post-germination stage in which the radicle begins to grow. Mobilization of reserves is one of the most critical events in germination, which could provide not only precursors but also energy for the biosynthetic processes. Although mobilization of the reserves may not be necessary for germination [11], it is crucial for germination efficiency and post-germinative seedling establishment [12].

Seed germination is a complex physiological process that is regulated by different external and internal factors, such as temperature [13], light [14], soil salinity [15], [16], gibberellic acid (GA) [17] and abscisic acid (ABA) [18]. During germination, the environmental and hormone signals integrate together to play regulatory roles [13], [19]. The environmental factors could affect the germination through the regulating the biosynthesis and catabolism of phytohormones, such as GA and ABA [20]. Some genes, such as embryonic identity genes LEAFY COTYLEDON1/LEAFYCOTYLEDON2/FUSCA3 (LEC1/LEC2/FUS3) and maternal gene *DNA binding with one zinc finger AFFECTINGGERMINATION* (DAG1/DAG2), are involved in the signaling of environmental factors or phytohormones and regulate the seed germination [21]. Regulation on reserves mobilization also happens. Based on previous studies, it is known that the phytohormone ABA helps to maintain seed dormancy [22], and hence inhibits reserves degradation. To the contrary, reserves degradation is promoted by GA. In cereal seeds, GA is synthesized during germination and induces the expression of alpha-amylase which promotes the degradation of starch [23]. Although there have been some studies about nutrient mobilization during seed germination [24], [25], how different reserves are mobilized, and how the mobilization is regulated during seed germination are largely unknown. To answer these questions, it is essential to explore the different pathways and their regulation mechanisms in different crops.

Network discovery, which is defined as elucidating the relationship between molecules and physiological or biochemical properties, is now achievable with the availability of large scale genomic information in many species [26]. The –omic strategies, such as transcriptomics and proteomics, have been proved to be powerful in constructing the metabolic networks. Owing to the complexity of seed germination, -omic strategies, especially proteomic methods have been widely used in studies of seed germination [22], [27]–[29]. Moreover, posttranslational modification behaviors of protein, which could only be studied through proteomic techniques, are also shown to be important for seed germination [30]–[32].

Previously, we have constructed metabolic and regulatory pathways in germinating rice seeds through proteome profiling [33]. Different from rice, soybean contains mainly storage oils and proteins, which makes it an ideal material to study the mobilization of reserves other than starches during germination. Furthermore, its genome has been sequenced [34]. Although many proteomic studies have been conducted on soybean [35], there are very few proteomic studies on its seed germination. To explore how different storage component were mobilized during germination, we carried out a comprehensive proteome profiling analysis on its germinating seeds. The proteome profile was then compared with that of rice germinating seeds. This work will help us to understand the distinct pathways for the reserves degradation and its regulation in different crops.

## Results and Discussion

### Germination process of rice and soybean seeds

**Figure 1:**
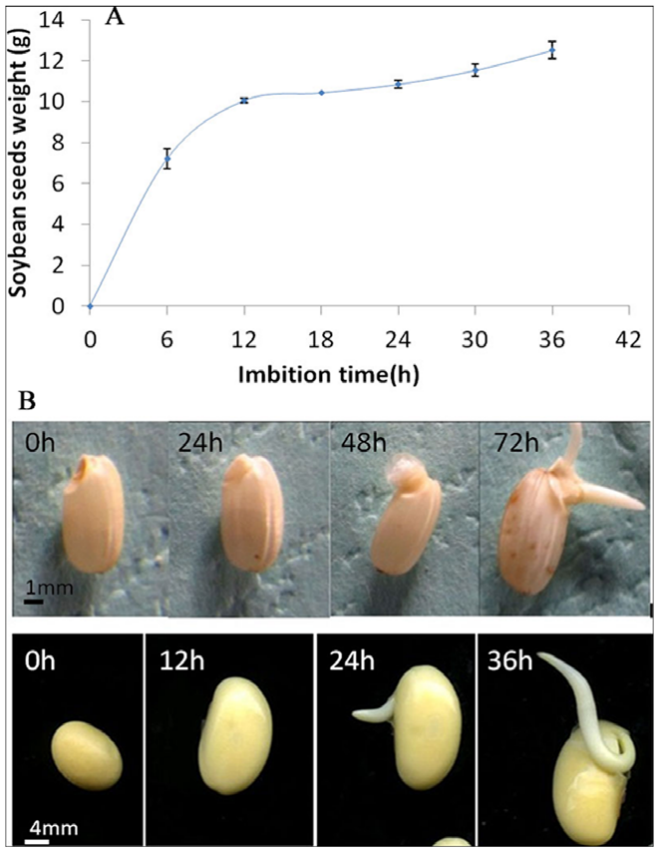
Germination process of soybean and rice seeds. A) water absorption curve during soybean seed germination; B) images of rice and soybean seeds during germination. Bars are 1 mm in rice images and 4 mm in soybean images.

Seed germination could be divided into three phases [6]. As reported, phase II of rice seed germination is the stage between 20 h and 50 h after imbibitions [36]. In order to compare with rice, proteome profiling in soybean should also be performed at the same stage, which requires definition of the time slot of its seed germination phases. In this study, phase II of soybean seeds germination was defined as 12–24 h after imbibition (Fig. 1A). At the end of this period, germination was almost complete (Fig. 1B). Based on this result, the germinating soybean seeds at 12 h after imbibition were used for further proteome profiling analysis. Compared with rice seed, it took less time to germinate (Fig. 1). It is known that rice seed has tiny embryo and belongs to morphological dormancy category. Its embryo has to grow inside the seed before protrusion [37], which is also supported by the observation that the embryo expands its size before radicle emergency (Fig. 1B).

### Proteome profile of germinating soybean seed

**Figure 2:**
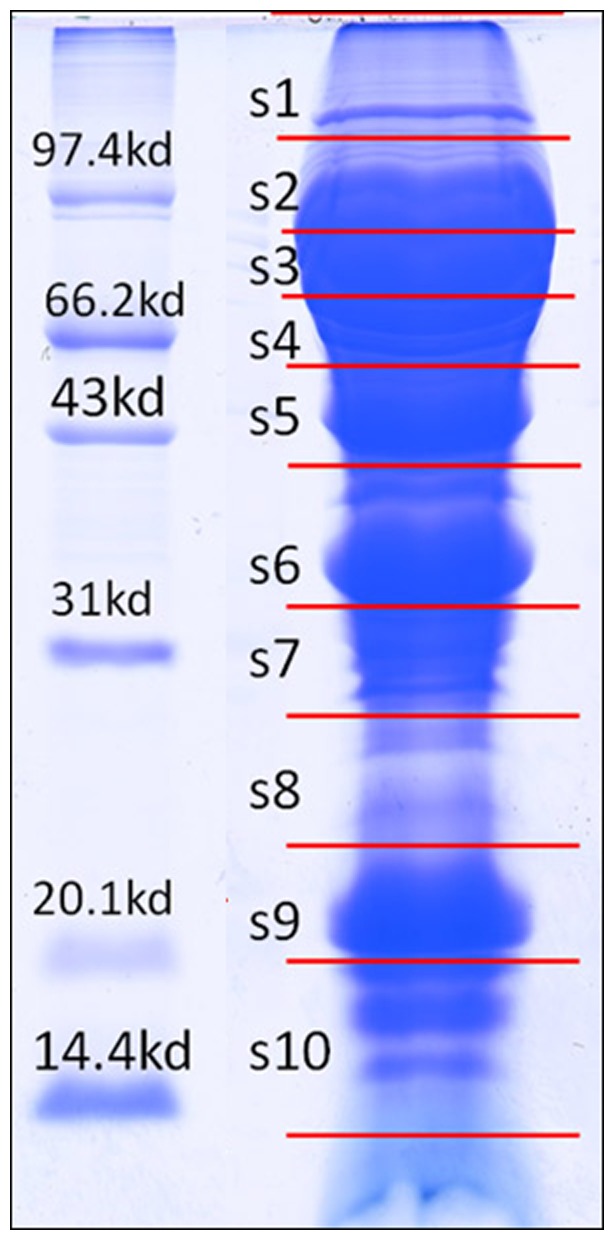
SDS-PAGE gel image of the germinating soybean seeds. 12% gel was used, and 4 ug of marker proteins were loaded. The s1–s10 indicate the 10 slices that were cut for in-gel digestion.

Proteins were extracted from soybean germinating seeds, and then subjected to one dimensional SDS-PAGE electrophoresis (1-DE). Figure 2 showed typical pattern of the gel image. For preparative electrophoresis, 40 ug proteins were loaded. After electrophoresis, the sample lane was cut into 10 slices (Fig. 2), and subjected to in-gel trypsin digestion, followed by liquid chromatography and tandem mass spectrometry (LC-MS/MS) analysis.

**Figure 3:**
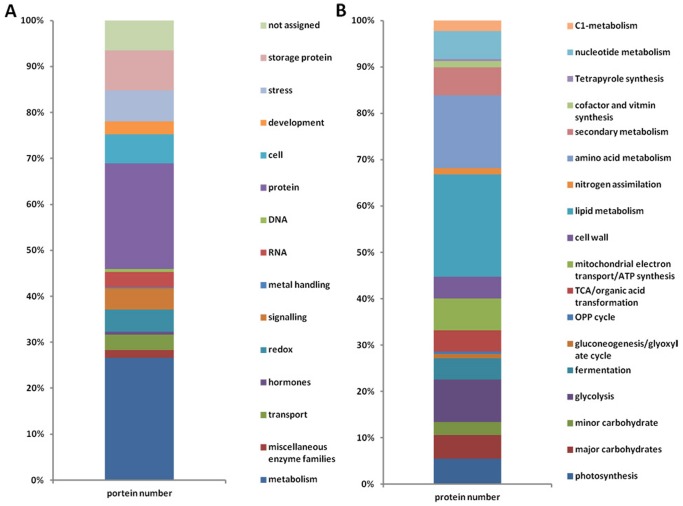
Functional categorization of the identified proteins. (A) total proteins; (B) the metabolism related proteins.

Totally, 764 non-redundant proteins were identified (Table S1, Table S2), slightly more than those identified in rice seeds [33]. However, there were 257 proteins that were annotated as putative or unknown proteins, which may be ascribed to the fact that the soybean is a newly sequenced species. These unknown proteins were blasted in NCBI database to obtain their homologs in other plant species. The best matches with probability >90% were selected. Based on this, 206 proteins from the putative/unknown proteins were assigned with different biological functions (Table S1). To better understand the physiological status of the imbibied seeds, the 764 identified proteins were classified into 14 functional categories (Fig. 3A) according to MapMan ontology. These categories are metabolism related proteins (215), protein biosynthesis and destination (183), RNA related proteins (28), DNA related proteins (5), redox regulation (36), stress response (55), signaling (38), transporting proteins (28), cell structure related proteins (48), development related proteins (22), storage proteins (33), hormone related (4), metal handling proteins (1), miscellaneous enzymes (15) and the function unassigned proteins (53).

The three categories with the most number of proteins are metabolism related proteins, proteins related to protein biosynthesis and destination and cell cycle and organization related proteins. Those for germinating rice seeds were metabolism, protein biosynthesis and destination and signaling proteins groups respectively [33]. However, the most abundant group is the storage proteins in soybean rather than the metabolism related proteins in rice. These proteins accounted for 42.8% of the total proteins in terms of abundance. Since proteins are the most abundant reserves in soybean seeds, it is reasonable that there are more storage proteins in soybean than in rice. The major storage proteins in soybean seed are 11S globulin (beta-conglycinin) and 7S globulin, whereas, those in rice are glutelin and globulin family proteins. It has been reported before that the degradation of these storage proteins might help to nourish the germinating soybean seeds and young seedlings [24]. Another obvious difference between the soybean and rice lists is the redox regulation proteins group. There were 36 redox regulation proteins identified in germinating soybean seeds, accounting for less than 0.5% of the total proteins in terms of abundance, whereas, 25 such proteins accounted for more than 8% in the germinating rice seeds. How the detoxification of ROS happened in soybean seed germination might be an interesting question.

### Metabolic pathways and characteristic of reserves mobilization

215 metabolic proteins were identified in germinating soybean seeds. To better understand the characteristics of reserves mobilization during soybean germination, these metabolic proteins were further categorized into 17 sub-groups based on the metabolic pathways they were involved in (Fig. 3B). These sub-groups include photosynthesis, major carbohydrates metabolism, glycolysis, fermentation, gluconeogenesis and glyoxylate cycle, phosphate pentose pathway, tricarboxylic acid (TCA) cycle, mitochondrial electron transport/ATP synthesis, cell wall metabolism, lipid metabolism, nitrogen metabolism, amino acid metabolism, secondary metabolism, cofactor and vitamin metabolism, tetrapyrrole synthesis, nucleotide metabolism and C1 metabolism (Table 1). There were 5 proteins that could be sorted into 2 or 3 different metabolic pathways (Table S1). Compared with the metabolic proteins identified in germinating rice seeds, proteins involved in phosphate pentose pathway, S-assimilation, and poly-amine synthesis were absent in soybean seeds (Table 1).

**Table 1:** Sub-categorization of the metabolism related proteins in germinating seeds of soybean and rice.

Functional class	Soybean	Rice
Photosynthesis	12	13
Major carbohydrate	10	23
Glycolysis	21	22
TCA cycle	10	15
Fermentation	10	6
Gluconeogenesis/glyoxylated cycle	2	5
Mitochondrial electron transport/ATP synthesis	15	8
Cell wall metabolism	10	8
Lipid metabolism	48	16
Nitrogen metabolism	3	1
Amino acid metabolism	34	47
Secondary metabolism	14	7
Cofactor and vitamin	3	2
nucleotide	13	15
C1 metabolism	6	3
Minor carbohydrates	6	6
Phosphate pentose pathway	0	6
OPP cycle	1	0
S-assimilation	0	1
Tetrapyrrole synthesis	1	2
Poly-amine synthesis	0	2

**Figure 4:**
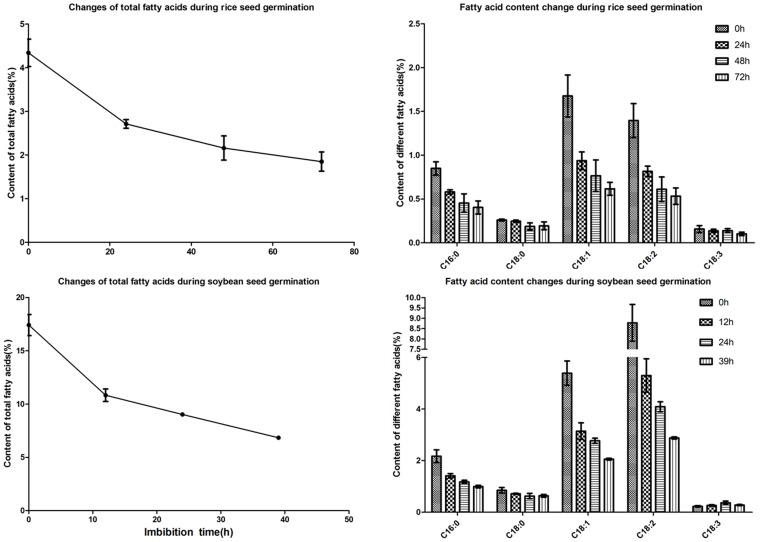
Dynamic changes of fatty acids during germination of soybean and rice seeds. (A) total fatty acids; (B) different compositions of fatty acids. Values were means of triplicate±SD.

Lipids and proteins are the two major reserves in soybean seeds. Mobilization of these two reserves should be very important for the soybean germination and the following seedling growth. This is supported by the fact that the lipid and amino acid metabolism related proteins were the two major sub-groups in the metabolic protein group (Fig. 3B). Fifty nine lipids metabolic proteins were detected (Table 1), much more than those detected in rice seeds. Most of the enzymes were lipoxygenase (LOX) (Table S1), which indicated that the oils in soybean seeds might be degraded through a LOX-dependent pathway. LOXs are non-heme iron-containing dioxygenases which catalyze the oxidation of polyunsaturated fatty acids by adding of molecular oxygen at C9 and C13 of the acyl chain in linolenic or linoleic acid [38]. It was reported that there were four major branches of LOX pathway: (a) The peroxygenase (POX) or hydroperoxide isomerase pathway, (b) The hydroperoxide dehydratase (AOS) pathway, (c) The hydroperoxide isomerase (HPL) pathway, (d) The divinyl ether (DES) pathway [39]. The detection of some P450 monooxygenases implied that the AOS branch might be the major pathway for lipids degradation during soybean germination. Some reports also showed that LOXs were storage proteins in soybean vegetative tissues and seeds, and might be associated with defense response [40]. Besides, LOXs and P450 monooxygenases, a full set of fatty acid beta-oxidation enzymes were also detected. The beta-oxidation along with glyoxylate cycle and TCA cycle will help to degrade the lipids completely. Compared with soybean, only 16 lipids metabolic proteins were detected in rice germinating seeds. We then measured the content of total fatty acids and their different compositions at different time points of seed germination in both soybean and rice. Although soybean seeds contained much more fatty acids than rice, the changes in fatty acids content were similar in these two crops (Fig. 4). The contents of fatty acid experienced a sharp decrease and then decreased slowly at the following stages (Fig. 4A). The results also showed that C18:1 and C18:2 were the major fatty acids in both rice and soybean seeds (Fig. 4B). The content of C18:2 fatty acids are much higher than C18:1 in soybean seeds, while more C18:1 fatty acids were detected in rice seeds (Fig. 4B).

**Figure 5:**
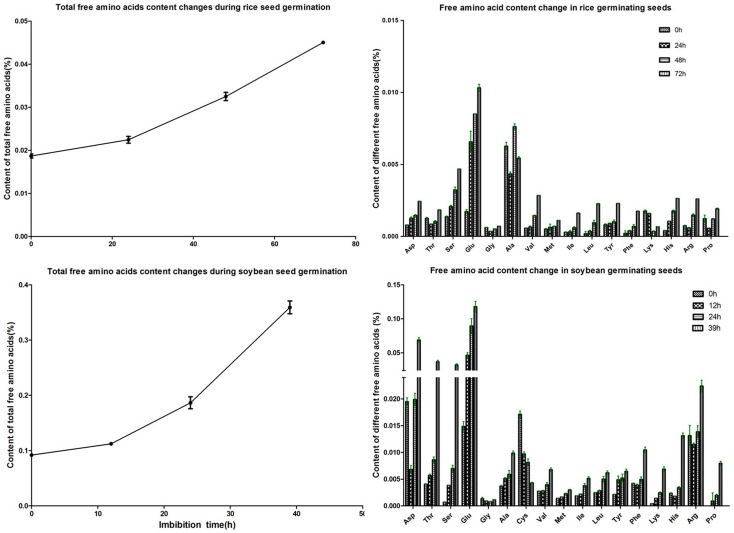
Dynamic changes of the free amino acids during germination of soybean and rice seeds. A) total free amino acids; B) different individual amino acids. Values were means of triplicate±SD.

There were 34 amino acid metabolism related proteins. Among them, methionine synthase and urease were the most abundant two. Methionine synthase catalyzes the final step of methionine biosynthesis, while urease catalyzes the degradation of arginine. This implied that the amino acids might be subject to different destinations during germination. The changes of free amino acids during germination were measured. The content of total free amino acids increased gradually along the germination (Fig. 5A). In general, there was an increase in all 20 amino acids (Fig. 5B). The degradation of proteins might be the source of free amino acids, since 55 protein-degradation related proteins were identified (Table S1). Similar results were also obtained in rice (Fig. 5). It was reported that the storage proteins might be degraded mainly through protease pathway in rice seed [29]. In soybean, protease C1 was reported as the enzyme that initiates the proteolysis of storage proteins during seed germination [41]. We did not detect this enzyme in our study. However, a lot of proteins belonging to 26s proteosome system were detected in soybean seed during germination, which indicated the 26 s proteosome degradation pathway might also play a role in this process.

**Figure 6:**
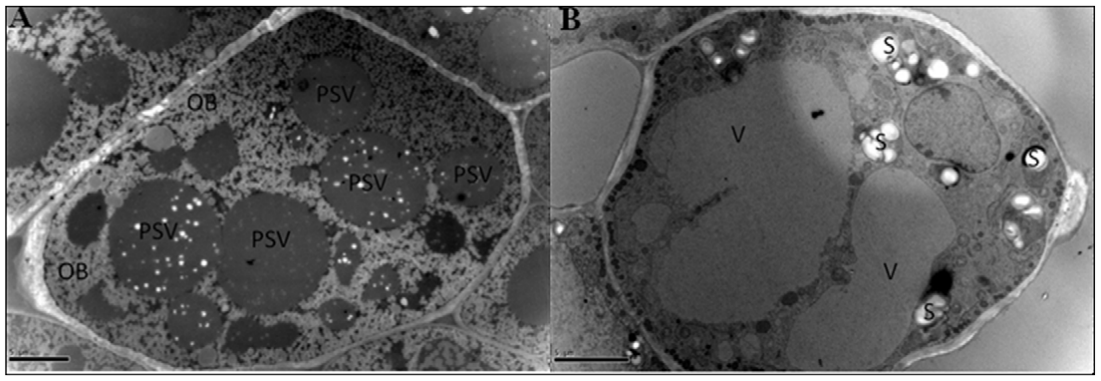
TEM images of soybean seeds showing the accumulation of starches at the late stage of soybean seed germination. (A) the dry seeds; (B) 3-day-imbibition seeds. PSV, protein stocking vesicle; OB, oil body; S, starch granule; V, vacuole. Bars' lengths are 5 um.

There were 10 major carbohydrates metabolism related proteins in germinating soybean seeds. Some starch catabolic enzymes such as beta-amylase, alpha-glucan water dikinase, endo-1,3-beta-glucanase and UDP-glucose 6-dehydrogenase were identified. Whereas, only one starch biosynthesis related enzymes ADP-glucose pyrophosphorylase was identified. There were 9 starch biosynthesis related proteins identified in germinating rice seed [29]. This difference may be ascribed to different reserves biosynthesis during seed filling in rice and soybean. In rice, starches were rapidly accumulated during seed maturation. So the enzymes were synthesized and could still function during germination. As for soybean seed, it mainly synthesizes proteins and lipids during seed maturation, so very few starch biosynthesis enzymes could be detected. These enzymes might be synthesized at the late stage of seed germination, because we could observe the accumulation of starch granules after 2 days imbibitions (Fig. 6). The accumulation of starch granules were also observed in both rice [29] and rapeseed (unpublished data) during germination. The conversion of different reserves into starch seems to be a routine process in different crops during seed germination. It is still an open question why plants sacrifice some ATPs to re-synthesize starches rather than directly utilize the reserves.

Although the enzymes involved in the degradation of lipids, proteins and amino acids, starches were all detected, it is more convincible to conclude that the soybean seeds mainly consume lipids at the early stage of germination with the comparison of their abundance.

### Redox regulation

**Figure 7:**
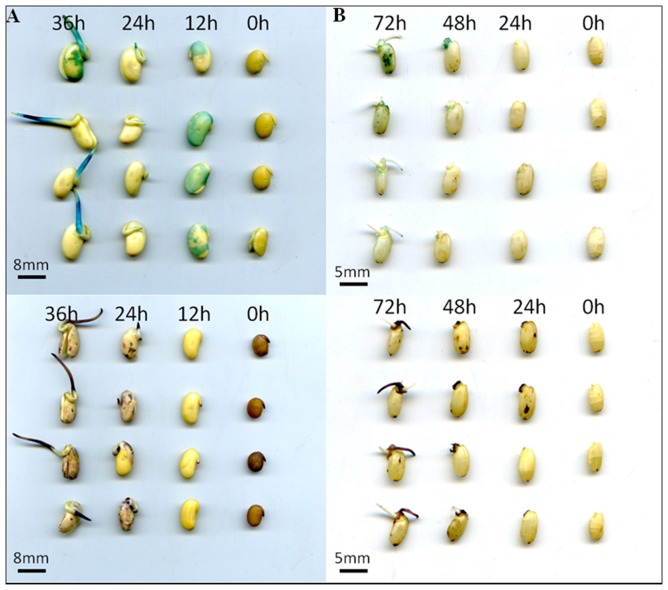
Accumulation of ROS in germinating crop seeds. (A) soybean; (B) rice. The upper panel shows the accumulation of H_2_O_2_ with TMB staining, the bottom panel shows the accumulation of superoxide anions.

Reactive oxygen species (ROS) can act as signal molecules to alleviate seed dormancy and promote seed germination [42]. However, over-accumulation of ROS might result in the oxidation of some physiologically important proteins or lipids and prevent germination [42], [43]. In soybean dry seeds, the ROS (mainly superoxide anion) were accumulated on the testa (Fig. 7A, bottom panel). These superoxide anions might be produced during storage and could be quickly converted into H_2_O_2_ within 12 hours upon imbibition (Fig. 7A, upper panel). Interestingly, the H_2_O_2_ on the testa could be hardly detected 24 hours after imbibitions (Fig. 7A). The H_2_O_2_ on testa might have either diffused into the water or been detoxified by the antioxidant proteins such as catalase and peroxidase. The accumulation of H_2_O_2_ was latter than superoxide anions, which is similar to that happened in rice seed germination (Fig. 7B) [29]. Totally, 36 redox regulatory proteins were identified in the germinating soybean seeds. However, these proteins just accounted less than 0.5% of the total proteins in terms of abundance. As mentioned above, LOXs can consume molecular oxygen when they function in lipids degradation and may help to release the oxidative stress for soybean seeds. This might be the reason that proteins involved in redox regulation were detected in very low abundance in soybean seeds. Among the 36 antioxidant proteins, glutathione S-transferase (GST), thioredoxin and peroxiredoxin were the three most abundant proteins. All of them can reduce the target proteins' disulfide and keep these proteins in their functional status [44], [45]. This indicates that soybean may prefer to protect the functional proteins from the attack of ROS during seed germination.

## Conclusions

Germination is the process during which the transition from physiologically quiescent to metabolically active status happens. The major metabolism happening is the mobilization of reserves, which provides not only energies but also precursors for the radicle emergence and seedling growth. In seeds of different crops, compositions of reserves are different. Proteins and oils are the two major reserves in soybean seeds, while starches are the predominant one for rice. Lipids might be mobilized through the LOXs dependent pathway during soybean seeds germination. Proteins were degraded through both protease and 26S proteosome system in soybean seeds, but through protease during rice seed germination. The LOXs could also function as ROS scavenger when reserves were rapidly mobilized during germination. The intermediates that were produced by the degradation of reserves might be transited into starches again and be utilized by the following seedling growth, which sounds to be a commonly phenomenon in different crops such as rice, soybean and rapeseed. These data may help us to get a comprehensive understanding of the physiological status and reserves mobilization mechanisms in crops seed germination.

## Methods

### Seed germination

Soybean (*Glycine max* “Zhongdou32”) seeds were shortly sterilized with 70% alcohol for 10 sec and then washed with distilled water three times with each for 5 min, and then imbibed in distilled water on soaked paper towel in the dark condition and 26°C. Seeds fresh weights were measured every 6 h until more than 90% of the seeds were germinated.

### Protein extraction and SDS-PAGE electrophoresis

Proteins were extracted from the germinating soybean seeds with acetone precipitation method. Briefly, 0.15–0.2 g germinating seeds were ground into fine powder in liquid nitrogen and homogenized with homogenization buffer containing 20 mM Tris/HCl (pH 7.5), 250 mM sucrose, 10 mM EGTA, 1 mM protease inhibitor cocktail (Sigma-Aldrich, Germany), 1 mM PMSF, 1 mM DTT, and 1% Triton X-100 and centrifuged at 15000 g (4°C) for 20 min, the supernatants were then collected and 3 volumes of cold acetone were added, and then incubated in −20°C for at least 2 h. The supernatant were then centrifuged, and the pellets were washed with cold acetone three times. The pellets were vacuum-dried and stored in −80°C for further use.

The protein powder was dissolved in lysis buffer through votexing for 1 h, and then added equal volume of 2×SDS loading buffer and boiled for 5 min. After centrifugation, supernatants were collected for polyacrylamide gel electrophoresis. For preparative running, 40 ug proteins were loaded onto 12% gel. After running, the gel was stained with Coomassie Brilliant Blue (CBB) R-250.

### Trypsin digestion and LC-MS/MS

The visualized gel was cut into 10 slices, each of which was then subjected to trypsin digestion and LC-MS/MS accroding to the method reported before [33]. Briefly, the gel was de-stained with de-staining solution (50 mM NH_4_HCO_3_ in 50% v/v ACN) until the gel was colorless, the gel pieces were rehydrated in 25 mM NH_4_HCO_3_ with 10 ng sequencing grade-modified trypsin (Promega, Madison, WI, USA) at 37°C overnight and the peptides were collected.

The peptides sample was desalted on RP trap columns (Zorbax 300 SB C18, Agilent Technologies), and were then separated on a RP column (150 µm i.d., 100 mm length, Column technology Inc., Fremont, CA). Mobile phase A (0.1% formic acid in HPLC-grade water) and the mobile phase B (0.1% formic acid in acetonitrile) were selected. 20 µg of tryptic peptide mixtures was loaded onto the columns, and separation was done at a flow rate of 2 µL/min by using a linear gradient of 4–50% B for 120 min. A FinniganTM LTQTM linear ion trap MS (Thermo Electron) equipped with an electrospray interface was connected to the LC setup for eluted peptides detection. Data-dependent MS/MS spectra were obtained simultaneously. Each scan cycle consisted of one full MS scan in profile mode followed by five MS/MS scans in centroid mode with the following Dynamic ExclusionTM settings: repeat count 2, repeat duration 30 s, exclusion duration 90 s. Each sample was analyzed in triplicate. MS/MS spectra were automatically searched against the non-redundant Viridiplantae taxon protein database using the BioworksBrowser rev. 3.1. The peptides were constrained to be tryptic and up to two missed cleavages were allowed. Carbamidomethylation of cysteines were treated as a fixed modification, whereas oxidation of methionine residue was considered as variable modifications. The mass tolerance allowed for the precursor ions was 2.0 Da and fragment ions was 0.0 Da, respectively. The output results were combined by BuildSummary software with a false positive rate (FPR) less than 1% as the filtration criterion. The FPR was calculated as double of the number of peptides from reversed database divided by the number of peptides from reversed and normal database [46]. The protein identification criteria were based on Delta CN (≥0.1) and cross-correlation scores (Xcorr, one charge≥1.9, two charges ≥2.2, three charges ≥3.75).

### Measurement of fatty acid, protein and free amino acids

About 0.01 g of seeds were ground in liquid nitrogen, and then added 2 ml methanol with 5% vitriol, 100 ul methanol with 0.2% BHT, 200 ul toluene and 15 ul C17:0 standard sample. This solution was esterified in 80°C for 3 h. Then, 1.5 ml 0.9% NaCl and 2 ml hexane with 0.2% BHT were added and vortexed for 2–3 min. After centrifuged at 2500 rpm for 2 min, the upper level solution was collected for GC. The fatty acid methyl esters were separated by Agilent 7890A GC system, fitted with 19091N-133 column (25 m, 0.25 mm, i.d). Nitrogen was used as carrier gas[47]. The sample was injured in split mode, FID (flame liquid ionization) temperature was 280°C and oven temperature was 180°C.

Free amino acids were extracted from seed with 80% ethanol three times. The collected supernatants were drained to remove water, and 0.02 M HCl was added to reach 1 ml final volume. Free amino acids were measured by Hitachi l-8800 amino acid analyzer.

### In situ staining of superoxide anion and hydrogen peroxide

Seeds were incubated in 6 mM NBT in 10 mM Tris-HCl buffer(pH 7.4) at room temperature for 30 min. Superoxide anion were visualized of dark-brown color [48]. Hydrogen peroxide were light-blue color after incubated in 0.42 mM TMB in Tris-acetate (pH 5.0) buffer for 1 h [49].

### Transmission electron microscopy (TEM) observation

For TEM analysis, cotyledon of soybean seed at different germinating stages were vacuum-infiltrated and pre-fixed in a solution of 2.5% glutaraldehyde which was adjusted to pH 7.4 with 0.1 M phosphate buffer, fixed in 2% OsO4 in the same buffer, and then dehydrated and embedded in epoxy resin and SPI-812 (Structure Probe, Inc). Ultra-thin sections obtained using a Leica UC6 ultramicrotome were stained with uranyl acetate and subsequently with lead citrate. The observations and recording of images were performed using a Hitachi H-7650 transmission electron microscope at 80 kV and a Gatan 832 CCD camera[50].

## Supporting Information

Table S1 **Proteins identified from germinating soybean seeds.**
(DOC)

Table S2 **Raw data of protein mass spectrometry identification.**
(XLSX)
