# Supplementary material for: Analysis of Proteome Profile in Germinating Soybean Seed, and Its Comparison with Rice Showing the Styles of Reserves Mobilization in Different Crops
Source: PLoS One. 2013 Feb 27;8(2):e56947. doi: 10.1371/journal.pone.0056947 (PMC3584108; doi:10.1371/journal.pone.0056947)
Supplement: Table S1 — Proteins identified from germinating soybean seeds. (DOC) [file pone.0056947.s001.doc]

Table S1 Proteins identified from germinating soybean seeds

| **Protein accession** | **Description** | **PC^a^** | **UPC^b^** | **SC^c^** | **MW(Da)** | **PI** |
| --- | --- | --- | --- | --- | --- | --- |
| **Metabolism** | | | | | | |
| **Photosynthesis (12)** | | | | | | |
| Q02909.1 | Phosphoenolpyruvate carboxylase, housekeeping isozyme | 8 | 6 | 8.89% | 110686.64 | 5.66 |
| ACJ38548.1 | ribulose-1,5-bisphosphate carboxylase/oxygenase large subunit | 10 | 6 | 16.49% | 52379.54 | 6.09 |
| P00865.1 | Ribulose bisphosphate carboxylase small chain 1 | 9 | 3 | 18.54% | 19962.03 | 8.87 |
| BAF43703.1 | Chlorophyllase 1 | 1 | 1 | 6.13% | 35967.21 | 7.16 |
| ACU23337.1 | senescence-inducible chloroplast stay-green protein 2 [Glycine max]* | 2 | 1 | 6.37% | 28646.98 | 8.1 |
| ACU23675.1 | gamma carbonic anhydrase 1 [Arabidopsis thaliana] | 2 | 1 | 4.81% | 29328.22 | 6.06 |
| ACU20371.1 | PREDICTED: probable ribose-5-phosphate isomerase-like [Glycine max]* | 1 | 1 | 4.98% | 29854.3 | 5.37 |
| CBG02743.1 | triosephosphate isomerase, putative [Ricinus communis]* | 36 | 13 | 70.75% | 27198 | 5.87 |
| CAY37271.1 | transketolase, putative [Ricinus communis]* | 4 | 1 | 4.79% | 78906.51 | 6.4 |
| CBG02769.1** | phosphoglycerate kinase, putative [Ricinus communis] | 4 | 1 | 4.75% | 57919.93 | 6.34 |
| ACU21329.1** | serine hydroxymethyltransferase 1 [Glycine max] | 5 | 4 | 12.90% | 48283.8 | 8.02 |
| ACU19625.1 | PREDICTED: fructose-bisphosphate aldolase 1, chloroplastic-like [glycine max]* | 1 | 1 | 6.22% | 25525.3 | 8.87 |
| **Major carbohydrate (10)** | | | | | | |
| CAL15435.1 | alpha-glucan water dikinase, chloroplast precursor, putative [Ricinus communis]* | 4 | 2 | 2.19% | 164159.31 | 6.45 |
| Q09WE7.1 | UDP-sugar pyrophosphorylase 1 | 2 | 1 | 1.83% | 66125.94 | 5.7 |
| ABC41950.1 | FAD-linked oxidoreductase 1 | 2 | 1 | 3.03% | 59736.76 | 7.28 |
| AAR26001.1 | endo-1,3-beta-glucanase | 2 | 2 | 5.21% | 57567.59 | 9.4 |
| CBD29723.1 | ADP-glucose pyrophosphorylase [Pisum sativum]* | 1 | 1 | 2.14% | 56271.39 | 6.23 |
| AAZ38832.1 | beta-amylase | 66 | 19 | 43.55% | 56071.7 | 5.33 |
| AAY40266.1 | beta-amylase | 43 | 17 | 40.12% | 56071.7 | 5.33 |
| Q96558.1 | UDP-glucose 6-dehydrogenase; Short=UDP-Glc dehydrogenase | 4 | 1 | 3.54% | 52941.68 | 5.74 |
| ACU21482.1 | glycogen phosphorylase, putative [Ricinus communis]* | 5 | 2 | 6.86% | 31474.03 | 6.26 |
| ACU21078.1 | PREDICTED: fructokinase-2-like [Glycine max]* | 1 | 1 | 17.06% | 18484.11 | 5 |
| **Minor carbonhydrate (6)** | | | | | | |
| ACU18683.1 | Aldose 1-epimerase family protein expressed [Medicago truncatula]* | 3 | 1 | 5.60% | 36954.54 | 6.12 |
| ACU18143.1 | PREDICTED: putative glucose-6-phosphate 1-epimerase-like isoform 1 [Glycine max]* | 1 | 1 | 4.31% | 36198.77 | 4.84 |
| ACU19687.1 | PREDICTED: putative glucose-6-phosphate 1-epimerase-like [Glycine max]* | 2 | 1 | 6.78% | 26520.39 | 7.63 |
| ACU20359.1 | PREDICTED: 2-dehydro-3-deoxyphosphooctonate aldolase-like [Glycine max]* | 2 | 1 | 3.78% | 31765.01 | 7.63 |
| ACU21192.1 | PREDICTED: carbohydrate kinase domain-containing protein [Vitis vinifera]* | 1 | 1 | 5.43% | 39635.59 | 8.28 |
| CBD32900.1 | NADPH-dependent mannose 6-phosphate reductase [Orobanche ramosa]* | 2 | 2 | 9.06% | 34714.69 | 5.93 |
| **Glycolysis (21)** | | | | | | |
| CBD33365.1 | 2,3-bisphosphoglycerate-independent phosphoglycerate mutase [Ricinus communis]* | 2 | 1 | 3.94% | 60840.08 | 5.51 |
| CBG02769.1** | phosphoglycerate kinase, putative [Ricinus communis] | 4 | 1 | 4.75% | 57919.93 | 6.34 |
| ACU18090.1 | Fructose-bisphosphate aldolase | 3 | 1 | 4.75% | 57919.93 | 6.34 |
| CBG02669.1 | putative pyruvate kinase [Arabidopsis thaliana]* | 2 | 1 | 4.75% | 57919.93 | 6.34 |
| AAS18240.1 | enolase | 13 | 7 | 21.95% | 50066.71 | 7.79 |
| Q42806.1 | Pyruvate kinase, cytosolic isozyme; | 1 | 1 | 4.95% | 47719.18 | 5.31 |
| CBG02669.1 | Pyruvate kinase [Oryza sativa Japonica Group]* | 3 | 1 | 2.94% | 55302.32 | 7.5 |
| ACU22850.1 | Fructose-bisphosphate aldolase | 14 | 7 | 24.10% | 47719.18 | 5.31 |
| AAS18240.1 | Enolase | 5 | 3 | 11.71% | 47719.18 | 5.31 |
| ACU21330.1 | Putative uncharacterized protein | 4 | 1 | 4.78% | 44673.75 | 8.56 |
| CBG02669.1 | putative pyruvate kinase [Arabidopsis thaliana]* | 1 | 1 | 7.22% | 42544.45 | 8.73 |
| ACU24103.1 | Fructose-bisphosphate aldolase | 18 | 9 | 35.41% | 42391.84 | 6.28 |
| CBG02768.1 | cytosolic phosphoglycerate kinase [Pisum sativum]* | 16 | 9 | 27.65% | 38339.59 | 7.12 |
| ACU24328.1 | glyceraldehyde 3-phosphate dehydrogenase [Nicotiana langsdorffii x Nicotiana sanderae]* | 38 | 7 | 31.18% | 36996.18 | 6.97 |
| ABC75834.1 | glyceraldehyde-3-phosphate dehydrogenase | 61 | 9 | 37.87% | 36763.95 | 6.72 |
| Q42806.1 | Pyruvate kinase, cytosolic isozyme | 9 | 6 | 32.09% | 28956.2 | 7.06 |
| CBG02744.1 | triosephosphate isomerase [Glycine max]* | 10 | 4 | 19.09% | 33160.77 | 6.35 |
| ACU23019.1 | Triosephosphate isomerase | 38 | 13 | 70.75% | 27204.01 | 5.87 |
| ACU20878.1 | glucose-6-phosphate isomerase, putative [Ricinus communis]* | 3 | 2 | 5.46% | 62672.45 | 5.9 |
| ACU17894.1 | UDP-glucose pyrophosphorylase [Amorpha fruticosa]* | 42 | 10 | 30.00% | 51493.97 | 5.41 |
| ACU18494.1 | UDP-glucose pyrophosphorylase [Amorpha fruticosa]* | 2 | 1 | 6.32% | 20755.43 | 5.05 |
| **Fermentation (10)** | | | | | | |
| CAV33689.1 | PREDICTED: alcohol dehydrogenase 1B-like [Glycine max]* | 2 | 1 | 3.51% | 45433.08 | 8.21 |
| CAY07289.1 | alcohol dehydrogenase 1 [Glycine max]* | 37 | 13 | 41.42% | 41184.21 | 6.39 |
| ACU18712.1 | alcohol dehydrogenase 1 [Glycine max]* | 49 | 16 | 50.13% | 41135.18 | 6.57 |
| CAY07290.1 | alcohol dehydrogenase-1F [Phaseolus acutifolius]* | 7 | 4 | 13.12% | 41052.07 | 5.98 |
| AAC62469.1 | alcohol dehydrogenase Adh-1 | 6 | 4 | 14.44% | 40241.15 | 6.09 |
| AAN03476.1 | alcohol dehydrogenase 1 | 35 | 14 | 47.41% | 40006.82 | 6.19 |
| AAC97495.1 | alcohol-dehydrogenase | 2 | 2 | 5.87% | 36380.58 | 6.13 |
| ACU19784.1 | alcohol dehydrogenase-1F [Phaseolus acutifolius]* | 4 | 2 | 7.93% | 31078.7 | 7.57 |
| ACU20757.1 | PREDICTED: alcohol dehydrogenase class-3-like [Glycine max]* | 6 | 2 | 11.61% | 40381.19 | 6.51 |
| ACU15185.1 | PREDICTED: alcohol dehydrogenase-like 2-like isoform 2 [Glycine max]* | 1 | 1 | 20.69% | 15846.22 | 5.35 |
| **Gluconeogenesis/glyoxylate cycle (2)** | | | | | | |
| ACU21405.1 | Isocitrate lyase and phosphorylmutase [Medicago truncatula]* | 7 | 5 | 29.43% | 31913.92 | 5.9 |
| P45458.1 | Malate synthase, glyoxysomal | 4 | 1 | 2.48% | 63909.22 | 7.02 |
| **TCA/organic acid transformation (10)** | | | | | | |
| CBC49069.1 | cytosolic malate dehydrogenase [Glycine max]* | 47 | 7 | 29.22% | 35467.77 | 5.91 |
| AAD56659.1 | malate dehydrogenase | 17 | 7 | 37.68% | 36141.68 | 8.23 |
| P37228.2 | Malate dehydrogenase, glyoxysomal; | 4 | 3 | 12.18% | 37394.56 | 7.55 |
| ACP19662.1 | aconitase | 1 | 1 | 6.50% | 22149.41 | 8.57 |
| AAC24855.1 | nodule-enhanced malate dehydrogenase | 3 | 2 | 7.26% | 43606.78 | 6.91 |
| ACU24596.1 | succinyl-CoA ligase alpha-chain 2 [Zea mays]* | 1 | 1 | 8.95% | 33791.07 | 8.98 |
| ACU20891.1 | nodule-enhanced malate dehydrogenase [Glycine max]* | 8 | 4 | 18.83% | 43147.28 | 8.11 |
| ACU17170.1 | PREDICTED: succinyl-CoA ligase [ADP-forming] subunit beta, mitochondrial [Glycine max] * | 1 | 1 | 6.40% | 18592.62 | 9.63 |
| ACU22728.1 | PREDICTED: ATP-citrate synthase alpha chain protein 1-like [Glycine max]* | 1 | 1 | 2.84% | 46561.78 | 5.57 |
| ACU13221.1 | PREDICTED: tropinone reductase homolog At1g07440-like isoform 1 [Glycine max]* | 1 | 1 | 4.02% | 27108.55 | 10 |
| **Opp cycle (1)** | | | | | | |
| ACU23896.1 | transaldolase-like protein [Solanum tuberosum]* | 8 | 3 | 9.34% | 48182.8 | 6.14 |
| **Mitochondrial electron transport/ATP synthesis (15)** | | | | | | |
| AAG28435.1 | AF195028_1 plasma membrane Ca2+-ATPase | 1 | 1 | 1.38% | 110740.14 | 5.66 |
| CAY05979.1 | mitochondrial ATPase beta subunit [Nicotiana sylvestris]* | 6 | 4 | 10.02% | 59836.22 | 5.8 |
| Q01915.1 | ATP synthase subunit alpha, mitochondrial* | 23 | 10 | 28.26% | 59836.22 | 5.8 |
| CAY05979.1 | mitochondrial ATPase beta subunit [Nicotiana sylvestris]* | 1 | 1 | 3.14% | 55753.93 | 5.15 |
| Q2PMS8.1 | ATP synthase subunit alpha, chloroplastic; | 21 | 9 | 24.80% | 55330.72 | 6.23 |
| ACJ38503.1 | membrane-bound ATP synthase subunit B | 1 | 1 | 3.83% | 50654.16 | 5.26 |
| ACU13679.1 | PREDICTED: ATP synthase subunit O, mitochondrial-like [Glycine max]* | 5 | 3 | 15.53% | 35324.35 | 9.14 |
| ACU18169.1 | ATP synthase gamma chain | 8 | 3 | 17.50% | 35197.31 | 9.34 |
| ACU24001.1 | Ubiquinol-cytochrome c reductase iron-sulfur subunit | 2 | 1 | 4.14% | 29306.62 | 8.87 |
| ACU17752.1 | ATP synthase gamma chain | 14 | 6 | 32.24% | 27027.45 | 9.47 |
| ACU19599.1 | PREDICTED: ATP synthase subunit O, mitochondrial-like [Glycine max]* | 14 | 6 | 34.69% | 26893.22 | 9.54 |
| CBD31408.1 | inorganic pyrophosphatase, putative [Ricinus communis]* | 2 | 1 | 8.45% | 24218.8 | 5.58 |
| ACU20795.1 | PREDICTED: ATP synthase subunit O, mitochondrial-like [Glycine max]* | 6 | 2 | 15.56% | 19948.61 | 4.7 |
| ACU23211.1 | PREDICTED: NADH dehydrogenase [ubiquinone] 1 alpha subcomplex subunit 9, mitochondrial-like [Glycine max]* | 4 | 3 | 10.09% | 47486.68 | 5.99 |
| ACU23274.1 | PREDICTED: probable ATP synthase 24 kDa subunit, mitochondrial-like [Glycine max]* | 4 | 3 | 22.41% | 27565.13 | 6.09 |
| **Cell wall (10)** | | | | | | |
| AAK01734.1 | chitinase class I | 3 | 1 | 6.88% | 34341.25 | 7.4 |
| ACU23018.1 | UDP-glucose:protein transglucosylase-like protein SlUPTG1 [Solanum lycopersicum]* | 3 | 2 | 10.33% | 41505.74 | 5.66 |
| ACU18769.1 | PREDICTED: UDP-arabinopyranose mutase 1-like [Glycine max]* | 2 | 1 | 5.32% | 40572.85 | 5.5 |
| ACU23724.1 | structural constituent of cell wall, putative [Ricinus communis]* | 1 | 1 | 6.48% | 31394.92 | 9.67 |
| ACU24580.1 | PREDICTED: UDP-glucose 4-epimerase GEPI48-like [Glycine max]* | 2 | 2 | 7.93% | 43023.09 | 7.66 |
| ACU18439.1 | UDP-glucose 4-epimerase [Pisum sativum]* | 10 | 5 | 18.29% | 39117.67 | 7.63 |
| ACU23582.1 | PREDICTED: UDP-glucuronic acid decarboxylase 1-like [Glycine max]* | 2 | 1 | 4.97% | 38568.23 | 6.68 |
| ACU22991.1 | PREDICTED: UDP-glucuronic acid decarboxylase 1-like [Glycine max]* | 1 | 1 | 10.96% | 33169.28 | 7.7 |
| CBD24878.1 | xyloglucan endotransglucosylase/hydrolase 3 [Cucumis melo]* | 1 | 1 | 4.56% | 32011.63 | 5.65 |
| ACU18251.1 | Xylose isomerase | 1 | 1 | 10.84% | 23318.86 | 8.2 |
| **Lipid metabolism (48)** | | | | | | |
| BAG09369.1 | peroxisomal acyl-CoA oxidase | 1 | 1 | 3.20% | 47878.41 | 8.88 |
| AAA81579.1 | acetyl-CoA carboxylase | 1 | 1 | 1.06% | 148945.5 | 6.24 |
| AAF61731.1 | beta-ketoacyl-ACP synthetase I-2 | 2 | 2 | 2.42% | 148945.5 | 6.24 |
| AAA33987.1 | lipoxygenase | 16 | 7 | 5.77% | 97251.83 | 5.99 |
| ABX60408.1 | lipoxygenase L-3 | 7 | 4 | 4.73% | 97251.83 | 5.99 |
| AAK20113.1 | Lipoxygenase | 167 | 32 | 49.48% | 97145.77 | 6.27 |
| AAA33987.1 | lipoxygenase | 1 | 1 | 2.31% | 97145.77 | 6.27 |
| 3BNB | A Chain A, Lipoxygenase-1 (Soybean) I553l Mutant | 51 | 7 | 8.33% | 96817.25 | 5.78 |
| ABS32275.1 | lipoxygenase-9 | 10 | 4 | 6.60% | 96817.25 | 5.78 |
| P08170.2 | Seed lipoxygenase-1 | 373 | 48 | 68.38% | 96798.29 | 6.12 |
| AAB41272.1 | lipoxygenase-3 | 3 | 3 | 6.88% | 96798.29 | 6.12 |
| ABS32275.1 | lipoxygenase-9 | 403 | 48 | 68.38% | 96768.27 | 6.12 |
| AAB67732.1 | lipoxygenase L-5 | 31 | 12 | 21.94% | 96768.27 | 6.12 |
| P24095.1 | Seed lipoxygenase | 206 | 30 | 46.67% | 96768.27 | 6.12 |
| ABX60408.1 | lipoxygenase L-3 | 23 | 13 | 23.10% | 96768.27 | 6.12 |
| 3BNB | Lipoxygenase-1 (Soybean) I553l Mutant | 312 | 45 | 66.24% | 96726.32 | 6.19 |
| AAB41272.1 | lipoxygenase-3 | 9 | 5 | 7.62% | 96657.95 | 5.63 |
| ABS32276.1 | lipoxygenase-10 | 4 | 2 | 2.57% | 96376.76 | 6.34 |
| AAB67732.1 | ipoxygenase L-5 | 31 | 10 | 10.17% | 96354.74 | 6.54 |
| AAK20113.1 | lipoxygenase | 10 | 5 | 5.78% | 96354.74 | 6.54 |
| AAA96817.1 | lipoxygenase | 6 | 3 | 4.77% | 96300.65 | 6.29 |
| ABX60411.1 | lipoxygease-like protein | 231 | 36 | 57.45% | 94411.53 | 5.91 |
| ABX60408.1 | lipoxygenase L-3 | 98 | 28 | 45.65% | 94411.53 | 5.91 |
| ABX60407.1 | lipoxygease L-2 | 21 | 7 | 13.23% | 94369.49 | 5.96 |
| P08170.2 | Full=Seed lipoxygenase-1 | 8 | 4 | 7.51% | 94369.49 | 5.96 |
| CBF60573.1 | acyl-CoA oxidase, putative [Ricinus communis]* | 2 | 1 | 1.81% | 74270.87 | 7.27 |
| AAA33988.1 | lipoxygenase-1 | 6 | 2 | 4.17% | 70552.21 | 8.86 |
| AAA96817.1 | lipoxygenase | 13 | 2 | 6.78% | 70293.56 | 5.07 |
| CBC76120.1 | glycerophosphoryl diester phosphodiesterase, putative [Ricinus communis]* | 1 | 1 | 2.08% | 68096.54 | 5.67 |
| BAB62890.1 | aspartic proteinase 1 | 4 | 2 | 6.03% | 55491.4 | 6.33 |
| ACU19360.1 | PREDICTED: 3-oxoacyl-[acyl-carrier-protein] reductase, chloroplastic-like [Glycine max]* | 1 | 1 | 2.77% | 49762.51 | 7.62 |
| AAA92462.1 | stearoyl-acyl carrier protein desaturase | 3 | 2 | 7.06% | 47282.79 | 5.8 |
| AAB00860.1 | microsomal omega-6 desaturase | 1 | 1 | 3.66% | 43967.89 | 8.74 |
| ACU23403.1 | PREDICTED: enoyl-[acyl-carrier-protein] reductase [NADH], chloroplastic-like [Glycine max]* | 6 | 3 | 14.03% | 41356.23 | 8.63 |
| P24095.1 | Seed lipoxygenase | 38 | 2 | 8.43% | 40539.57 | 5.8 |
| ABS32276.1 | lipoxygenase-10 | 9 | 2 | 8.43% | 40539.57 | 5.8 |
| CBC57217.1 | GDSL esterase/lipase [Medicago truncatula]* | 1 | 1 | 2.78% | 39575.21 | 8.42 |
| BAA09852.1 | Epoxide hydrolase | 5 | 3 | 14.66% | 39168.95 | 5.64 |
| AAA81579.1 | acetyl-CoA carboxylase | 4 | 2 | 9.88% | 36403.71 | 6.36 |
| ACU21519.1 | peroxisomal 3-ketoacyl-CoA thiolase [Glycine max]** | 6 | 3 | 18.62% | 36384.47 | 8.89 |
| ABB85235.1 | malonyltransferase | 4 | 1 | 7.19% | 33734.57 | 9.17 |
| ACU23629.1 | PREDICTED: (3R)-hydroxymyristoyl-[acyl-carrier-protein] dehydratase-like [Glycine max]* | 2 | 1 | 6.36% | 24366.65 | 9.58 |
| ACU22847.1 | PREDICTED: enoyl-[acyl-carrier-protein] reductase [NADH], chloroplastic-like [Glycine max] * | 7 | 2 | 5.42% | 49514.99 | 9.2 |
| ABC68416.1 | cytochrome P450 monooxygenase CYP74A1 | 1 | 1 | 2.46% | 54876.98 | 8.3 |
| ABD97099.1 | cytochrome P450 monooxygenase CYP94D24 | 1 | 1 | 2.46% | 54876.98 | 8.3 |
| ABC68409.1 | cytochrome P450 monooxygenase CYP90A15 | 1 | 1 | 4.27% | 40293.59 | 9.27 |
| ABC68416.1 | cytochrome P450 monooxygenase CYP74A1 | 2 | 1 | 3.52% | 39367.23 | 9.25 |
| ACU23057.1 | PREDICTED: enoyl-CoA hydratase 2, peroxisomal-like [Glycine max]* | 2 | 1 | 6.86% | 19145.78 | 6.05 |
| **Nitrogen assimilation (3)** | | | | | | |
| AAB50233.1 | nitrite reductase | 1 | 1 | 1.85% | 66911.95 | 6.95 |
| ACU21191.1 | haloacid dehalogenase-like hydrolase domain-containing protein [Arabidopsis thaliana]* | 1 | 1 | 4.98% | 26699.55 | 5.56 |
| ACU18102.1 | cytosolic glutamine synthetase alpha [Glycine max]* | 1 | 1 | 7.02% | 39212.07 | 5.22 |
| **Amino acid metabolism (34)** | | | | | | |
| ACU23012.1 | PREDICTED: phospho-2-dehydro-3-deoxyheptonate aldolase 1, chloroplastic-like [Glycine max]* | 1 | 1 | 9.04% | 18529.35 | 9.29 |
| CAY04052.1 | Glycine dehydrogenase [decarboxylating], mitochondrial | 1 | 1 | 1.42% | 114907.79 | 7.2 |
| AAO85884.1 | embryo-specific urease | 62 | 18 | 31.74% | 90156.75 | 5.68 |
| ABC00741.1 | threonine synthase | 1 | 1 | 4.27% | 56326.24 | 6.68 |
| BAG09377.1 | peroxisomal betaine-aldehyde dehydrogenase | 2 | 2 | 4.97% | 54739.89 | 5.23 |
| BAG09376.1 | peroxisomal betaine-aldehyde dehydrogenase | 2 | 1 | 3.78% | 54611.01 | 5.35 |
| ACU18663.1 | PREDICTED: diaminopimelate decarboxylase 1, chloroplastic-like [Glycine max]* | 2 | 1 | 2.05% | 54058.73 | 6.32 |
| ABW17196.1 | alanine aminotransferase 1 | 2 | 2 | 4.94% | 53320.78 | 5.32 |
| CBG02726.1 | argininosuccinate synthase, putative [Ricinus communis]* | 4 | 2 | 5.66% | 52574.39 | 6.5 |
| ACU18522.1 | PREDICTED: argininosuccinate synthase, chloroplastic-like [Glycine max]* | 3 | 2 | 5.88% | 52361.86 | 6.54 |
| ACU21329.1** | serine hydroxymethyltransferase 1 [Glycine max] | 5 | 4 | 12.90% | 48283.8 | 8.02 |
| ACU21446.1 | PREDICTED: fumarylacetoacetase-like [Glycine max]* | 7 | 3 | 12.59% | 45867.32 | 5.95 |
| AAC50015.1 | aspartate aminotransferase cytosolic isozyme AAT2 | 12 | 7 | 21.96% | 45600.27 | 7.72 |
| ACU18134.1 | PREDICTED: bifunctional aspartate aminotransferase and glutamate/aspartate-prephenate aminotransferase-like [Glycine max]* | 2 | 1 | 4.05% | 43669.23 | 5.56 |
| BAG09371.1 | peroxisomal 3-hydroxyisobutyryl-coenzyme A hydrolase | 1 | 1 | 5.68% | 42943.41 | 8.88 |
| ACU23158.1 | BBMII isomerase [Arabidopsis lyrata subsp. lyrata]* | 2 | 1 | 5.90% | 41429.92 | 6.68 |
| P93164.1 | Gamma-Glu-X carboxypeptidase | 7 | 3 | 11.40% | 37676.48 | 6.08 |
| CBG02692.1 | cysteine synthase [Glycine max]* | 3 | 2 | 9.85% | 34400.97 | 5.53 |
| CBG07435.1 | cysteine synthase [Glycine max]* | 1 | 1 | 5.23% | 34269.8 | 5.69 |
| ACU18801.1 | PREDICTED: ATP phosphoribosyltransferase-like [Glycine max]* | 1 | 1 | 4.67% | 32499.15 | 5.66 |
| ACU20307.1 | chorismate mutase [Populus trichocarpa]* | 1 | 1 | 6.71% | 32249.92 | 5.45 |
| ACU17605.1 | PREDICTED: indole-3-glycerol phosphate lyase, chloroplastic [Glycine max]* | 2 | 1 | 5.62% | 28236.71 | 5.53 |
| AAG34872.1 | In2-1 protein | 17 | 7 | 32.07% | 27031 | 5.21 |
| ACU18650.1 | Cysteine synthase | 2 | 1 | 7.85% | 26000.14 | 7.61 |
| AAT94362.1 | putative chalcone isomerase 4 | 4 | 4 | 37.80% | 23495.73 | 4.89 |
| AAK69432.1 | chalcone isomerase | 6 | 4 | 24.77% | 23264.85 | 6.23 |
| AAQ22725.1 | amino acid aminotransferase | 1 | 1 | 5.88% | 18993.56 | 7.13 |
| ACU14594.1 | methionine synthase [Glycine max]* | 1 | 1 | 6.13% | 18472.51 | 9.3 |
| AAT94363.1 | chalcone isomerase 4B | 2 | 2 | 23.36% | 15650.89 | 4.64 |
| ACU18413.1 | Ni-binding urease accessory protein UreG [Glycine max]* | 2 | 1 | 7.37% | 31409.12 | 5.99 |
| CBD28044.1 | methionine synthase [Glycine max]* | 53 | 18 | 34.34% | 84282.27 | 5.93 |
| CBD28028.1 | methionine synthase [Glycine max]* | 56 | 20 | 40.37% | 84271.38 | 5.97 |
| ACU19183.1 | PREDICTED: putative lactoylglutathione lyase-like [Glycine max]* | 4 | 1 | 5.00% | 31646.02 | 5.37 |
| ACU18122.1 | PREDICTED: glyoxylate reductase-like [Glycine max]* | 1 | 1 | 12.46% | 34285.57 | 5.93 |
| CBC96810.1** | cytosolic acetoacetyl-coenzyme A thiolase [Nicotiana tabacum] | 1 | 1 | 5.90% | 41556.51 | 6.16 |
| ACU21519.1** | peroxisomal 3-ketoacyl-CoA thiolase [Glycine max] | 6 | 3 | 18.62% | 36384.47 | 8.89 |
| **Secondary metabolism (14)** | | | | | | |
| AAF17578.1 | isoflavone reductase homolog 2 | 12 | 7 | 36.13% | 33939.6 | 5.6 |
| CBD32724.1 | dihydroflavonol reductase [Glycine max]* | 3 | 2 | 9.03% | 34959.41 | 5.65 |
| AAT47734.1 | isoflavone synthase 2 | 3 | 2 | 5.89% | 59362.46 | 9.17 |
| P26690.1 | NAD(P)H-dependent 6'-deoxychalcone synthase | 8 | 4 | 20.63% | 35489.85 | 6.32 |
| ACU22699.1 | 2-hydroxyisoflavanone dehydratase | 1 | 1 | 6.58% | 35138.14 | 5.75 |
| CBC51434.1 | S-adenosyl-L-methionine: caffeic acid 3-0-methyltransferase [Glycine max]* | 1 | 1 | 3.56% | 39997.38 | 5.85 |
| ACU19602.1 | PREDICTED: probable caffeoyl-CoA O-methyltransferase At4g26220-like isoform 1 [Glycine max]* | 1 | 1 | 6.30% | 26693.74 | 5.62 |
| CBC51426.1 | PREDICTED: caffeic acid 3-O-methyltransferase-like [Glycine max]* | 1 | 1 | 4.57% | 41622.9 | 5.26 |
| CBC96810.1** | cytosolic acetoacetyl-coenzyme A thiolase [Nicotiana tabacum] | 1 | 1 | 5.90% | 41556.51 | 6.16 |
| ACU24017.1 | PREDICTED: isopentenyl-diphosphate Delta-isomerase II-like [Glycine max]* | 1 | 1 | 0.0465 | 34081.81 | 5.78 |
| ACU18155.1 | PREDICTED: isopentenyl-diphosphate Delta-isomerase II-like [Glycine max]* | 11 | 7 | 29.41% | 27372.21 | 5.13 |
| CAZ77889.1** | tropinone reductase [Cochlearia officinalis] | 7 | 4 | 23.22% | 28431.2 | 5.77 |
| ACU19301.1** | PREDICTED: tropinone reductase homolog At1g07440-like isoform 1 [Glycine max] | 4 | 3 | 19.33% | 28966.83 | 5.99 |
| ACU23577.1 | PREDICTED: strictosidine synthase-like [Glycine max]* | 1 | 1 | 3.72% | 38845.4 | 7.65 |
| **Cofactor and vitamin synthesis (4)** | | | | | | |
| ACU19865.1 | Ubiquinone biosynthesis protein coq-8, putative [Ricinus communis]* | 1 | 1 | 11.76% | 15650.73 | 9.64 |
| ACU24561.1 | PREDICTED: ubiquinone biosynthesis protein COQ9, mitochondrial-like [Glycine max]* | 4 | 3 | 18.21% | 32223.51 | 8.39 |
| AAY85184.1 | dehydroascorbate reductase | 2 | 2 | 11.58% | 28863.3 | 8.54 |
| ACU20290.1** | gamma-glutamyl hydrolase precursor [Glycine max] | 8 | 3 | 20.33% | 26922.6 | 5.43 |
| **Tetrapyrole synthesis (1)** | | | | | | |
| CAX69281.1 | chlorophyllide a oxygenase [Arabidopsis thaliana]* | 1 | 1 | 3.20% | 60443.48 | 7.65 |
| **Nucleotide metabolism (13)** | |  |  |  |  |  |
| ACU13653.1 | PREDICTED: adenine phosphoribosyltransferase 1, chloroplastic-like [Glycine max]* | 5 | 2 | 14.41% | 25531.84 | 8.62 |
| AAO23951.1 | phosphoribosylformylglycinamidine synthase | 2 | 1 | 1.07% | 143519.45 | 4.95 |
| ACU18635.1 | phosphoribosylamine--glycine ligase [Glycine max]* | 1 | 1 | 3.25% | 45073.37 | 4.86 |
| AAL18815.1 | inosine-5'-monophosphate dehydrogenase-like protein | 3 | 2 | 6.89% | 41871.65 | 6.69 |
| ACU19171.1 | PREDICTED: adenosine kinase 2-like [Glycine max]* | 4 | 2 | 9.38% | 37587.83 | 5.5 |
| ACU21508.1 | PREDICTED: adenosine kinase 2 [Vitis vinifera]* | 1 | 1 | 6.16% | 37443.96 | 5.29 |
| P04670.2 | Uricase-2 isozyme 1; | 8 | 7 | 30.42% | 35138.06 | 7.74 |
| ACU21289.1 | Adenylate kinase [Medicago truncatula] | 1 | 1 | 6.34% | 31320.92 | 8.96 |
| ACU19250.1 | PREDICTED: adenylate kinase B-like [Glycine max]* | 3 | 2 | 11.98% | 26625.89 | 7.65 |
| ACU14249.1 | Nucleoside diphosphate kinase | 10 | 3 | 21.48% | 16439.78 | 6.84 |
| AAN77500.1 | nucleoside diphosphate kinase | 15 | 5 | 32.89% | 16354.77 | 6.91 |
| AAN77501.1 | nucleoside diphosphate kinase | 13 | 4 | 27.03% | 16341.64 | 6.3 |
| CBD31415.1 | soluble inorganic pyrophosphatase [Malus x domestica]* | 1 | 1 | 8.23% | 26124.99 | 5.51 |
| **C1-metabolism (6)** | | | | | | |
| ACU18647.1 | formate dehydrogenase [Phaseolus vulgaris]* | 50 | 10 | 37.89% | 42823.8 | 6.28 |
| ACU19857.1 | formate dehydrogenase [Phaseolus vulgaris]* | 52 | 10 | 40.42% | 41880.93 | 6.9 |
| ACU21329.1** | serine hydroxymethyltransferase 1 [Glycine max] | 5 | 4 | 12.90% | 48283.8 | 8.02 |
| ACU24251.1 | PREDICTED: c-1-tetrahydrofolate synthase, cytoplasmic-like [Glycine max]* | 14 | 5 | 22.45% | 31490.53 | 7.72 |
| ACU23686.1 | PREDICTED: c-1-tetrahydrofolate synthase, cytoplasmic-like [Glycine max]* | 15 | 6 | 26.87% | 31457.48 | 8.31 |
| ACU18271.1 | short chain dehydrogenase, putative [Ricinus communis]* | 46 | 11 | 50.51% | 31726.02 | 7.59 |
| **Miscellaneous enzyme families ()** | | | | | | |
| CBD06075.1 | PREDICTED: UDP-glycosyltransferase 85A7-like [Glycine max]* | 1 | 1 | 3.74% | 51428.13 | 6.68 |
| ACU23709.1 | PREDICTED: endo-1,3;1,4-beta-D-glucanase-like [Glycine max]* | 2 | 1 | 6.91% | 23602.33 | 8.94 |
| ACU18052.1 | epoxide hydrolase [Glycine max]* | 1 | 1 | 3.46% | 35944.06 | 5.42 |
| CBD28487.1 | beta-glucosidase, putative [Ricinus communis]* | 3 | 3 | 15.03% | 36492.43 | 5.85 |
| ACU19478.1 | epoxide hydrolase [Glycine max]* | 2 | 2 | 7.28% | 35769.93 | 5.93 |
| ACU19184.1 | PREDICTED: dehydrogenase/reductase SDR family member 7-like [Glycine max]* | 1 | 1 | 3.76% | 35085.95 | 9.3 |
| ACU18023.1 | Hydroxysteroid 11-beta-dehydrogenase 1-like protein [Medicago truncatula]* | 1 | 1 | 3.10% | 40157.46 | 6.22 |
| CBG18415.1 | short chain dehydrogenase, putative [Ricinus communis]* | 102 | 12 | 60.54% | 31660.95 | 6.39 |
| ACU19769.1 | quinone oxidoreductase [Fragaria x ananassa]* | 1 | 1 | 6.27% | 34583.66 | 6.03 |
| ACU21266.1 | PREDICTED: tropinone reductase homolog [Glycine max]* | 5 | 2 | 12.55% | 29047.93 | 6.9 |
| ACU19301.1 | PREDICTED: tropinone reductase homolog At1g07440-like isoform 1 [Glycine max]** | 4 | 3 | 19.33% | 28966.83 | 5.99 |
| CAZ77889.1** | tropinone reductase [Cochlearia officinalis] | 7 | 4 | 23.22% | 28431.2 | 5.77 |
| ACU23905.1 | Dehydrogenase/reductase SDR family member [Medicago truncatula]* | 1 | 1 | 5.10% | 27095.4 | 9.15 |
| ACU17500.1 | PREDICTED: tropinone reductase homolog isoform 1 [Glycine max]* | 3 | 2 | 11.32% | 22950.41 | 8.51 |
| CAZ77880.1 | short chain dehydrogenase [Solanum tuberosum]* | 4 | 1 | 8.68% | 27595.17 | 6.75 |
| **Metal handling (1)** | | | | | | |
| ACU23060.1 | aluminum induced protein with YGL and LRDR motifs [Arabidopsis thaliana]* | 9 | 5 | 30.51% | 25364.83 | 5.77 |
| **Transporting (28)** | | | | | | |
| ACU14166.1 | Outer membrane lipoprotein blc [Medicago truncatula] * | 5 | 3 | 22.28% | 21311.02 | 5.83 |
| CBD23688.1 | nitrate transporter NRT1-5 [Glycine max]* | 1 | 1 | 1.85% | 65446.45 | 8.94 |
| AAB03894.1 | glucose binding protein | 155 | 28 | 51.34% | 60522.78 | 6.42 |
| AAF05723.1 | sucrose binding protein homolog S-64 | 92 | 22 | 44.99% | 55834.47 | 6.32 |
| AAO48716.1 | sucrose-binding protein 2 | 88 | 22 | 41.92% | 55775.41 | 6.12 |
| CAC67472.1 | selenium binding protein | 5 | 3 | 8.58% | 53064.01 | 5.62 |
| ACU19295.1 | Sec61 transport protein [Populus trichocarpa]* | 1 | 1 | 2.52% | 52150.23 | 8.96 |
| ACU18379.1 | PREDICTED: ADP,ATP carrier protein 3, mitochondrial-like [Glycine max]* | 7 | 3 | 11.02% | 39709.56 | 9.77 |
| ACU24498.1 | PREDICTED: translocase of chloroplast 34-like [Glycine max]* | 2 | 1 | 7.37% | 34816.29 | 9.35 |
| ACU20451.1 | secretory carrier membrane protein, putative [Ricinus communis]* | 2 | 1 | 3.92% | 34171.92 | 8.81 |
| ACU19524.1 | PREDICTED: alpha-soluble NSF attachment protein-like [Glycine max]* | 3 | 2 | 11.76% | 32603.92 | 5.06 |
| AAA33983.1 | lectin prepeptide | 33 | 4 | 22.81% | 30927.93 | 5.65 |
| ACU24178.1 | PREDICTED: outer plastidial membrane protein porin-like isoform 1 [Glycine max]* | 7 | 2 | 14.49% | 29815.63 | 8.57 |
| ACU21099.1 | PREDICTED: mitochondrial outer membrane protein porin of 36 kDa-like [Glycine max]* | 8 | 4 | 17.39% | 29789.32 | 7.07 |
| ACU18944.1 | ferritin-2, chloroplastic [Glycine max]* | 12 | 4 | 22.78% | 29170.05 | 5.94 |
| ACU23390.1 | PREDICTED: putative mitochondrial 2-oxoglutarate/malate carrier protein-like [Glycine max]* | 2 | 2 | 13.19% | 29167.92 | 9.38 |
| 1SBF-A | Agglutinin SBA | 179 | 16 | 70.36% | 27571.91 | 5.15 |
| ACU21541.1 | PREDICTED: nascent polypeptide-associated complex subunit alpha | 4 | 1 | 9.70% | 26167.82 | 4.81 |
| ACU21104.1 | Transmembrane emp24 domain-containing protein 10 precursor, putative [Ricinus communis]* | 1 | 1 | 5.43% | 25351.06 | 6.06 |
| ACU24317.1 | GDP dissociation inhibitor [Cicer arietinum]* | 3 | 2 | 13.45% | 24157.47 | 4.28 |
| AAA33958.1 | ferritin light chain | 3 | 2 | 7.08% | 23924.98 | 5.4 |
| ABB02392.1 | temperature-induced lipocalin | 3 | 2 | 9.78% | 21446.22 | 6.62 |
| ACU14659.1 | Outer membrane lipoprotein blc [Medicago truncatula]* | 3 | 2 | 13.04% | 21207.96 | 5.76 |
| 3A9Q | Ferritin-4, chloroplastic | 4 | 2 | 16.40% | 20927.69 | 5.68 |
| BAG09387.1 | peroxisomal voltage-dependent anion-selective channel protein | 6 | 2 | 22.10% | 19400.85 | 8.71 |
| CBD23677.1 | PREDICTED: extended synaptotagmin-3-like [Glycine max]* | 1 | 1 | 4.64% | 61751.66 | 6.28 |
| ACU13727.1 | Mitochondrial import inner membrane translocase subunit Tim17/Tim22/Tim23 family protein [Arabidopsis thaliana]* | 4 | 2 | 20.99% | 18854.26 | 5.91 |
| ACU18088.1 | PREDICTED: uncharacterized mitochondrial carrier C12B10.09-like [Glycine max]* | 2 | 1 | 5.80% | 22354.96 | 9.62 |
| **Redox (36)** | | | | | | |
| 3KAL-B | Homoglutathione synthetase | 1 | 1 | 3.21% | 55660.69 | 5.95 |
| O48561.1 | Catalase-4 | 1 | 1 | 3.46% | 56737.08 | 6.8 |
| O48560.1 | Catalase-3 | 2 | 1 | 3.46% | 56911.24 | 6.77 |
| CBC66228.1 | glutathione S-transferase GST 21 [Glycine max]* | 2 | 1 | 9.17% | 24611.18 | 5.36 |
| CBC22919.1 | glutathione S-transferase GST 14 [Glycine max]* | 3 | 2 | 8.29% | 25535.72 | 8.87 |
| CBC22906.1 | glutathione S-transferase GST 14 [Glycine max]* | 11 | 5 | 25.89% | 25889.96 | 6.24 |
| ACU20577.1 | Superoxide dismutase [Cu-Zn] | 1 | 1 | 10.08% | 13732.9 | 5.9 |
| ACU16940.1 | Superoxide dismutase | 6 | 3 | 19.17% | 26575.23 | 8.59 |
| ACU14800.1 | Glutathione peroxidase | 1 | 1 | 5.99% | 18502.9 | 6.59 |
| ABQ52658.1 | Superoxide dismutase | 7 | 3 | 19.09% | 26706.39 | 8.56 |
| AAQ13492.1 | iron-superoxide dismutase | 8 | 4 | 20.49% | 27466.16 | 5.45 |
| AAG34811.1 | glutathione S-transferase GST 21 | 2 | 1 | 9.71% | 23388.62 | 5.16 |
| ACU17741.1 | protein disulfide isomerse like protein [Glycine max]* | 2 | 1 | 4.13% | 47486.68 | 5.99 |
| ACU23175.1 | protein disulfide isomerse like protein [Glycine max]* | 4 | 3 | 10.09% | 47486.68 | 5.99 |
| ACU13514.1 | PREDICTED: thioredoxin-like 3-3-like [Glycine max]* | 3 | 3 | 12.09% | 40491.08 | 5.45 |
| BAG16715.1 | protein disulfide isomerase L-2 | 4 | 2 | 4.95% | 58590.91 | 5.13 |
| BAG16714.1 | protein disulfide isomerase | 5 | 2 | 7.08% | 61611.68 | 4.69 |
| BAD24712.1 | protein disulfide isomerase-like protein | 3 | 2 | 6.90% | 61611.68 | 4.69 |
| ACU14710.1 | Thioredoxin | 47 | 13 | 42.67% | 58715.05 | 5.06 |
| CBD16504.1 | glutaredoxin [Litchi chinensis]* | 1 | 1 | 13.43% | 14858.91 | 5.93 |
| ACU14895.1 | peroxiredoxin [Pisum sativum]* | 24 | 5 | 38.27% | 17453.17 | 5.41 |
| ACU15753.1 | mitochondrial peroxiredoxin [Pisum sativum]* | 1 | 1 | 8.12% | 21181.18 | 8.77 |
| ACU20981.1 | PREDICTED: 2-Cys peroxiredoxin BAS1-like, chloroplastic-like [Glycine max]* | 3 | 3 | 30.05% | 22331.44 | 5.93 |
| ACU19072.1 | PREDICTED: 1-Cys peroxiredoxin-like [Glycine max]* | 9 | 6 | 40.37% | 24420.05 | 6.44 |
| AAR26529.1 | glutathione S-transferase 2 | 1 | 1 | 5.13% | 26463.36 | 5.1 |
| CBC66360.1 | putative glutathione S-transferase [Phaseolus acutifolius]* | 2 | 1 | 5.02% | 25653.6 | 5.57 |
| CBC22871.1 | glutathione S-transferase [Glycine max]* | 7 | 4 | 20.55% | 25578.6 | 5.73 |
| CBC22936.1 | glutathione S-transferase [Phaseolus acutifolius]* | 1 | 1 | 7.66% | 25502.62 | 6.13 |
| CBC22934.1 | glutathione S-transferase [Phaseolus acutifolius]* | 8 | 3 | 13.43% | 25085.03 | 5.59 |
| CBC22880.1 | glutathione S-transferase [Populus alba x Populus tremula var. glandulosa]* | 2 | 2 | 10.23% | 24772.55 | 5.74 |
| CBC22926.1 | glutathione S-transferase [Cucurbita maxima]* | 1 | 1 | 8.13% | 23878.6 | 5.28 |
| ACU17496.1 | PREDICTED: monothiol glutaredoxin-S17-like [Glycine max]* | 2 | 1 | 9.38% | 21768.9 | 5.25 |
| ACU14052.1 | dehydroascorbate reductase [Malpighia glabra]* | 21 | 10 | 62.44% | 23488.16 | 5.81 |
| ACU13726.1 | glutathione S-transferase GST 22 [Glycine max]* | 29 | 8 | 48.57% | 27636.72 | 5.72 |
| ACU18627.1 | PREDICTED: monodehydroascorbate reductase-like isoform 1 [Glycine max] | 3 | 3 | 12.25% | 43580.79 | 5.88 |
| ACU23343.1 | NADH-cytochrome b5 reductase-like protein [Medicago truncatula]* | 1 | 1 | 3.76% | 35079.4 | 8.63 |
| **Hormones (4)** | | | | | | |
| CAM82580.1 | 1-aminocyclopropane-1-carboxylate deaminase, putative [Ricinus communis]* | 1 | 1 | 5.28% | 41461.58 | 8.22 |
| CBD26841.1 | PREDICTED: 12-oxophytodienoate reductase 2-like [Glycine max]* | 2 | 1 | 4.59% | 41316.71 | 6.36 |
| CBC72168.1 | PREDICTED: 1-aminocyclopropane-1-carboxylate oxidase 1-like [Glycine max]* | 1 | 1 | 6.51% | 34808.68 | 5.42 |
| ACU19449.1 | PREDICTED: probable indole-3-acetic acid-amido synthetase GH3.6-like [Glycine max]* | 1 | 1 | 2.60% | 64974.79 | 5.64 |
| ACU14456.1 | methionine synthase [Glycine max]* | 1 | 1 | 12.35% | 19750.78 | 5.85 |
| **Signaling (38)** | | | | | | |
| ACJ37405.1 | receptor-like protein kinase | 1 | 1 | 3.53% | 75196.73 | 8.29 |
| CBD02400.1 | cbl-interacting protein kinase 10 [Arabidopsis lyrata subsp. lyrata]* | 1 | 1 | 2.16% | 52361.22 | 8.77 |
| ACT80135.1 | PREDICTED: interferon-related developmental regulator 1-like [Glycine max]* | 1 | 1 | 4.68% | 49427.03 | 6.74 |
| CBC30929.1 | fiber annexin [Gossypium hirsutum]* | 3 | 2 | 7.91% | 35922.88 | 6.48 |
| CBD34855.1 | annexin-like protein [Arachis hypogaea var. vulgaris]* | 15 | 8 | 28.80% | 35922.88 | 6.48 |
| CBD34855.1 | annexin-like protein [Arachis hypogaea var. vulgaris]* | 15 | 7 | 19.23% | 35877.7 | 7.72 |
| CBC30932.1 | annexin [Medicago truncatula]* | 23 | 12 | 42.95% | 35877.7 | 7.72 |
| CBC30929.1 | annexin, putative [Ricinus communis]* | 3 | 3 | 8.95% | 35765.8 | 6.79 |
| ACU23023.1 | guanine nucleotide-binding protein subunit beta-like protein [Glycine max]* | 15 | 9 | 32.62% | 35748.27 | 7.62 |
| CBD34851.1 | annexin-like protein [Medicago sativa]* | 7 | 3 | 9.15% | 35506.53 | 8.85 |
| ACU24279.1 | Type 2A phosphatase activator TIP41, putative [Ricinus communis]* | 1 | 1 | 4.81% | 33137.6 | 5.04 |
| CBD05667.1 | putative protein phosphatase 2C 39 [Arabidopsis thaliana]* | 6 | 3 | 13.48% | 30791.89 | 5.87 |
| AAB09583.1 | SGF14D | 13 | 6 | 22.99% | 29518.16 | 4.79 |
| CBD34946.1 | 14-3-3-like protein [Cicer arietinum]* | 15 | 9 | 34.35% | 29354.01 | 4.67 |
| AAB09582.1 | SGF14C | 11 | 7 | 29.07% | 29208.78 | 4.72 |
| CBD09250.1 | 14-3-3 protein [Vigna angularis]* | 19 | 10 | 34.75% | 29161.76 | 4.66 |
| ACU24102.1 | inositol monophosphatase [Phaseolus vulgaris]* | 2 | 2 | 15.19% | 29114.06 | 4.99 |
| ACU21069.1 | inositol monophosphatase [Phaseolus vulgaris]* | 5 | 4 | 25.93% | 29081.97 | 4.85 |
| CBD09255.1 | 14-3-3 family protein [Malus x domestica]* | 18 | 9 | 44.36% | 29047.82 | 4.71 |
| Q96451.1 | 14-3-3-like protein B | 7 | 5 | 28.34% | 27921.49 | 4.75 |
| AAB71227.1 | Ca+2-binding EF hand protein | 21 | 8 | 40.59% | 26979.66 | 5.98 |
| ACU21290.1 | PREDICTED: probable calcium-binding protein CML21-like [Glycine max]* | 3 | 1 | 5.68% | 26014.54 | 4.75 |
| CBC86128.1 | calcineurin B-like protein [Phaseolus vulgaris]* | 1 | 1 | 6.19% | 25681.1 | 4.62 |
| ACN76808.1 | AIR12 | 3 | 1 | 8.61% | 25640.98 | 6.96 |
| ACU20637.1 | GTP-binding nuclear protein Ran-1 [Arabidopsis thaliana]* | 3 | 2 | 9.50% | 25299.92 | 6.5 |
| ACU23007.1 | PREDICTED: ras-related protein RABC1 isoform 1 [Vitis vinifera]* | 1 | 1 | 5.14% | 23798.1 | 5.69 |
| Q43463.1 | Ras-related protein Rab7 | 2 | 2 | 10.65% | 23786.72 | 6.22 |
| ACU24038.1 | small GTP-binding protein [Pisum sativum]* | 2 | 1 | 7.01% | 23601.77 | 8.37 |
| ACU24372.1 | PREDICTED: ras-related protein RABA1f-like [Glycine max]* | 3 | 1 | 8.96% | 23481.49 | 4.66 |
| ACU13427.1 | PREDICTED: ras-related protein Rab7-like isoform 1 [Glycine max]* | 2 | 2 | 12.32% | 23169.33 | 7.69 |
| AAA90955.1 | guanine nucleotide regulatory protein | 4 | 2 | 11.65% | 23110.26 | 5.46 |
| AAA34003.1 | Rab7p | 5 | 3 | 22.82% | 23052 | 5.09 |
| ACU24291.1 | PREDICTED: ras-related protein Rab11C-like [Glycine max]* | 7 | 2 | 17.41% | 22386.43 | 5.15 |
| AAD17207.1 | ADP-ribosylation factor | 8 | 6 | 44.38% | 20351.25 | 6.42 |
| ACU17433.1 | PREDICTED: ras-related protein RABD2a-like [Glycine max]* | 2 | 1 | 18.81% | 11556.03 | 4.96 |
| ACU18790.1 | rab-type small GTP-binding protein [Cicer arietinum]* | 2 | 2 | 23.86% | 9992.34 | 5.74 |
| ABF65987.1 | CETS1 | 2 | 1 | 9.88% | 19039.1 | 9.1 |
| CBD31728.1 | mitogen-activated protein kinase kinase MAPKK2 [Glycine max]* | 1 | 1 | 3.37% | 39697.54 | 5.25 |
| **RNA and DNA (33)** | | | | | | |
| **RNA (28)** | | | | | | |
| ACU14617.1 | PREDICTED: regulator of ribonuclease-like protein 3-like [Glycine max]* | 3 | 3 | 24.70% | 17754.22 | 6.29 |
| Q8HVY3.2 | DNA-directed RNA polymerase subunit beta | 1 | 1 | 1.15% | 159057.3 | 9.52 |
| BAG72096.1 | Gag-protease-integrase-RT-RNaseH polyprotein | 1 | 1 | 0.68% | 150213.27 | 8.87 |
| ABQ42349.1 | trihelix transcription factor | 2 | 1 | 2.00% | 56817.21 | 6.39 |
| AAP85545.1 | putative WRKY-type DNA binding protein | 1 | 1 | 3.25% | 53572.16 | 6.84 |
| ACU18744.1 | transcription termination factor family protein [Arabidopsis thaliana]* | 1 | 1 | 3.67% | 53292.98 | 8.96 |
| CBC40946.1 | DNA methyltransferase 1-associated protein 1 [Arabidopsis thaliana]* | 1 | 1 | 1.62% | 48843.88 | 9.46 |
| ACU24233.1 | PREDICTED: SURP and G-patch domain-containing protein 1-like protein-like [Glycine max]* | 1 | 1 | 3.00% | 47721.52 | 8.65 |
| ABC47854.1 | bzip transcription factor | 1 | 1 | 2.64% | 45245.66 | 9.81 |
| ACU20788.1 | PREDICTED: zinc finger protein ZAT9-like [Glycine max]* | 1 | 1 | 2.58% | 43352.82 | 5.07 |
| ACU19563.1 | transcription factor [Glycine max]* | 1 | 1 | 4.39% | 35881.61 | 5.55 |
| CBD33201.1 | RNA-binding protein [Glycine max]* | 2 | 1 | 4.35% | 32913.77 | 4.85 |
| ACU17732.1 | PREDICTED: probable rRNA-processing protein EBP2 homolog [Glycine max]* | 1 | 1 | 2.09% | 32701.64 | 9.05 |
| CBC57022.1 | AP2 domain class transcription factor [Malus x domestica]* | 1 | 1 | 4.91% | 31525.27 | 4.78 |
| AAL65125.1 | GT-2 | 1 | 1 | 3.12% | 27158.99 | 9.16 |
| CBC01814.1 | NAC domain class transcription factor [Malus x domestica]* | 1 | 1 | 5.70% | 26077.48 | 9.35 |
| ACU20588.1 | PREDICTED: G patch domain-containing protein 8-like [Glycine max]* | 1 | 1 | 7.77% | 22348.05 | 8.92 |
| ACU14783.1 | PREDICTED: nascent polypeptide-associated complex subunit alpha-like protein-like [Glycine max]* | 6 | 3 | 22.17% | 22129.35 | 4.43 |
| CBC85816.1 | putative quinone oxidoreductase [Cicer arietinum]* | 2 | 2 | 19.51% | 21760.79 | 6.09 |
| CBC85820.1 | benzoquinone reductase [Gossypium hirsutum]* | 2 | 2 | 15.27% | 21738.9 | 6.43 |
| ACU13403.1 | PREDICTED: malignant T cell-amplified sequence 1-like [Glycine max]* | 1 | 1 | 11.05% | 19939.32 | 8.72 |
| ACU13185.1 | RNA polymerase II transcriptional coactivator KELP [Arabidopsis thaliana]* | 1 | 1 | 10.43% | 18923.23 | 5.12 |
| ACU16880.1 | putative MYC protein [Tamarix hispida]* | 1 | 1 | 7.64% | 17852.28 | 6.12 |
| CAI94204.1 | cellular nucleic acid-binding protein [Arabidopsis thaliana]* | 2 | 1 | 8.89% | 17431.45 | 6.29 |
| ACU15744.1 | PREDICTED: transcription initiation factor TFIID subunit 10-like [Glycine max]* | 1 | 1 | 8.09% | 15261.1 | 5.22 |
| ACU18048.1 | PREDICTED: auxin response factor 3-like [Glycine max]* | 1 | 1 | 11.67% | 13552.53 | 9.73 |
| ACU15943.1 | Histone deacetylase complex subunit SAP18 [Medicago truncatula]* | 1 | 1 | 4.64% | 16777.31 | 9.44 |
| ACU21122.1 | transcription factor [Vicia faba var. minor]* | 1 | 1 | 3.54% | 39382.91 | 5.96 |
| **DNA (5)** | | | | | | |
| P22177.1 | Proliferating cell nuclear antigen | 1 | 1 | 5.08% | 26134.7 | 4.65 |
| CBD21806.1 | Helicase, C-terminal [Medicago truncatula]* | 12 | 6 | 20.58% | 46771.49 | 5.45 |
| AAG59996.1 | ferredoxin:sulfite reductase precursor | 1 | 1 | 2.27% | 63821.9 | 9.14 |
| ACU19951.1 | DNA-damage-repair/toleration protein-like[oryza sativa]* | 4 | 2 | 10.00% | 32725.94 | 5.28 |
| CBC72156.1 | Flavonol synthase[Medicago truncatula]* | 1 | 1 | 2.56% | 39472.41 | 5.33 |
| **Protein (183)** | | | | | | |
| **Amino acid activation (4)** | | | | | | |
| ACU22713.1 | methionyl-tRNA formyltransferase, putative [Ricinus communis]* | 1 | 1 | 4.53% | 38231.25 | 9.21 |
| CAV26500.1 | glutaminyl-tRNA synthetase, putative [Ricinus communis]* | 3 | 2 | 5.28% | 90148.37 | 5.88 |
| ACU23233.1 | asparaginyl-tRNA synthetase, cytoplasmic 1 [Arabidopsis thaliana]* | 2 | 1 | 6.79% | 25699.8 | 4.88 |
| ACU18731.1 | tyrosyl-tRNA synthetase, putative [Ricinus communis] * | 1 | 1 | 3.68% | 43277.88 | 6.17 |
| **Synthesis (95)** | | | | | | |
| CBC52171.1 | PREDICTED: peptidyl-prolyl cis-trans isomerase CYP19-3-like [Glycine max]* | 1 | 1 | 10.29% | 18955.56 | 8.33 |
| Q43467.1 | Elongation factor Tu, chloroplastic | 14 | 7 | 19.02% | 49405.98 | 9.14 |
| P62302.1 | 40S ribosomal protein S13 | 5 | 3 | 20.00% | 23347.35 | 10.2 |
| P46280.1 | Elongation factor Tu, chloroplastic | 7 | 5 | 16.91% | 52509.12 | 6.33 |
| P25698.2 | Elongation factor 1-alpha | 14 | 6 | 20.53% | 47587.68 | 5.92 |
| P18663.2 | 50S ribosomal protein L2-A, chloroplastic | 12 | 3 | 28.91% | 14099.4 | 10.07 |
| O22518.1 | 40S ribosomal protein SA | 2 | 1 | 6.67% | 13947.19 | 9.83 |
| CBD35496.1 | translation elongation factor-1 alpha [Oryza sativa Japonica Group]* | 12 | 7 | 19.02% | 49377.88 | 9.14 |
| CBD33106.1 | eukaryotic translation initiation factor 2c, putative [Ricinus communis]* | 3 | 2 | 10.03% | 31719.18 | 5.13 |
| CBD32482.1 | 60S ribosomal protein L10, putative [Ricinus communis]* | 4 | 3 | 26.51% | 17788.61 | 9.06 |
| CBD31846.1 | S18.A ribosomal protein [Arabidopsis thaliana]* | 8 | 3 | 26.97% | 17500.27 | 10.56 |
| CBD31845.1 | S18.A ribosomal protein [Jatropha curcas]* | 7 | 5 | 22.22% | 29705.4 | 9.81 |
| CBD28176.1 | ribosomal protein S14 [Elaeis guineensis]* | 3 | 2 | 12.50% | 28109.91 | 10.65 |
| CBD21909.1 | 40S ribosomal protein S3, putative [Ricinus communis]* | 4 | 1 | 17.39% | 10210.93 | 10.3 |
| CBD21906.1 | 40S ribosomal protein S3, putative [Ricinus communis]* | 4 | 3 | 20.32% | 20879.62 | 11.17 |
| CBD12010.1 | 60S ribosomal protein L10a-1 [Zea mays]* | 2 | 2 | 18.05% | 15673.43 | 10.81 |
| CBC91827.1 | 60S ribosomal protein L35a [Vernicia fordii]* | 4 | 2 | 19.33% | 16294.72 | 10.87 |
| CBC77190.1 | 40S ribosomal protein SA [Glycine max]* | 1 | 1 | 6.50% | 14319.24 | 10.81 |
| CBC15122.1 | translation elongation factor 1A-2 [Gossypium hirsutum]* | 9 | 6 | 19.21% | 52095.45 | 6.21 |
| ACU24626.1 | PREDICTED: 40S ribosomal protein S4-1-like [Glycine max]* | 6 | 3 | 25.38% | 14929.37 | 10.92 |
| ACU24606.1 | PREDICTED: 60S ribosomal protein L11-like [Glycine max]* | 6 | 2 | 16.91% | 23735.74 | 10.93 |
| ACU24584.1 | Elongation factor 1, gamma chain; Glutathione S-transferase, C-terminal; Thioredoxin-like fold [Medicago truncatula]* | 8 | 5 | 15.79% | 47527.7 | 5.77 |
| ACU24574.1 | Elongation factor 1, gamma chain; Glutathione S-transferase, C-terminal; Thioredoxin-like fold [Medicago truncatula]* | 14 | 7 | 19.02% | 49279.82 | 9.15 |
| ACU24496.1 | Ribosomal protein L15 | 2 | 2 | 20.54% | 12258.31 | 9.51 |
| ACU24072.1 | PREDICTED: 50S ribosomal protein L5, chloroplastic-like [Glycine max]* | 5 | 2 | 13.82% | 24732.29 | 10.43 |
| ACU24044.1 | PREDICTED: 60S ribosomal protein L26-1-like [Glycine max]* | 35 | 9 | 47.03% | 26306.74 | 9.59 |
| ACU23898.1 | PREDICTED: 40S ribosomal protein S3a-like [Glycine max]* | 2 | 2 | 15.83% | 13815.2 | 11.6 |
| ACU23780.1 | 60S ribosomal protein L6 [Cicer arietinum]* | 1 | 1 | 14.66% | 22208.77 | 9.82 |
| ACU23780.1 | 60S ribosomal protein L6 [Cicer arietinum]* | 10 | 3 | 29.68% | 17401.53 | 10.41 |
| ACU23742.1 | PREDICTED: elongation factor 1-beta 1-like [Glycine max]* | 6 | 3 | 16.14% | 24304.44 | 4.58 |
| ACU23723.1 | 40S ribosomal protein S6 | 8 | 4 | 29.30% | 17628.82 | 10.21 |
| ACU23653.1 | 60S ribosomal protein L13 | 3 | 1 | 6.31% | 23711.25 | 10.28 |
| ACU23653.1 | 60S ribosomal protein L13 | 7 | 3 | 26.97% | 17513.3 | 10.67 |
| ACU23594.1 | ribosomal protein L5, putative [Ricinus communis]* | 2 | 1 | 6.92% | 14774.3 | 9.89 |
| ACU23400.1 | PREDICTED: eukaryotic translation initiation factor 4E-2 [Glycine max]* | 1 | 1 | 1.61% | 116911.21 | 9.41 |
| ACU23062.1 | PREDICTED: 60S ribosomal protein L9-like isoform 1 [Glycine max] * | 7 | 3 | 29.14% | 17140.11 | 10.37 |
| ACU23062.1 | PREDICTED: 60S ribosomal protein L9-like isoform 1 [Glycine max]* | 6 | 2 | 24.82% | 16013.67 | 10.31 |
| ACU21516.1 | PREDICTED: 40S ribosomal protein S3a-like [Glycine max]* | 2 | 2 | 15.15% | 15400.28 | 10.42 |
| ACU21407.1 | PREDICTED: 60S ribosomal protein L6-like [Glycine max]* | 2 | 2 | 11.43% | 15012.68 | 10.4 |
| ACU21251.1 | PREDICTED: 40S ribosomal protein S19-1-like [Glycine max]* | 3 | 1 | 6.28% | 23735.74 | 10.93 |
| ACU20951.1 | 40S ribosomal protein S6 | 4 | 3 | 23.56% | 21997.54 | 9.75 |
| ACU20858.1 | 60S acidic ribosomal protein PO [Euphorbia esula]* | 11 | 5 | 30.31% | 34158.25 | 5.15 |
| ACU20582.1 | PREDICTED: 40S ribosomal protein S5-like [Glycine max]* | 3 | 2 | 21.01% | 13521.49 | 9.57 |
| ACU20562.1 | PREDICTED: 40S ribosomal protein S9-2-like [Glycine max]* | 5 | 4 | 27.08% | 21717.14 | 9.52 |
| ACU20345.1 | 40S ribosomal protein S6 | 2 | 1 | 8.15% | 15732.25 | 9.98 |
| ACU19839.1 | PREDICTED: 40S ribosomal protein S23-like isoform 1 [Glycine max]* | 2 | 1 | 6.47% | 25901.35 | 10.2 |
| ACU19508.1 | PREDICTED: 60S ribosomal protein L30-like [Glycine max]* | 8 | 4 | 18.77% | 29639.31 | 9.81 |
| ACU19438.1 | 60S ribosomal protein L32, putative [Ricinus communis] * | 4 | 1 | 6.86% | 24192.96 | 11.58 |
| ACU19424.1 | putative eukaryotic translation initiation factor 1 [Citrus sinensis]* | 1 | 1 | 6.50% | 22708.55 | 5.71 |
| ACU19332.1 | PREDICTED: 60S ribosomal protein L7a-like [Glycine max]* | 5 | 3 | 15.00% | 29636.19 | 10.19 |
| ACU19026.1 | PREDICTED: 60S ribosomal protein L7-4-like [Glycine max]* | 1 | 1 | 9.77% | 15571.34 | 10.7 |
| ACU18370.1 | 40S ribosomal protein S8 | 6 | 3 | 25.58% | 14812.28 | 10.92 |
| ACU17578.1 | PREDICTED: 60S ribosomal protein L27a-3-like [Glycine max]* | 3 | 1 | 8.84% | 16556.42 | 10.59 |
| ACU17122.1 | PREDICTED: 40S ribosomal protein S16-like [Glycine max]* | 4 | 2 | 12.85% | 28255.04 | 10.7 |
| ACU17053.1 | PREDICTED: 60S ribosomal protein L12-like [Glycine max]* | 7 | 4 | 24.50% | 34093.01 | 9.45 |
| ACU17029.1 | PREDICTED: 60S ribosomal protein L8-3 [Glycine max]* | 2 | 2 | 6.31% | 23587.12 | 10.33 |
| ACU16822.1 | PREDICTED: 40S ribosomal protein S7 [Glycine max]* | 8 | 4 | 26.04% | 21701.16 | 9.58 |
| ACU16774.1 | PREDICTED: 40S ribosomal protein S7 [Glycine max]* | 1 | 1 | 14.66% | 22342.91 | 9.76 |
| ACU16612.1 | PREDICTED: 40S ribosomal protein S26-1-like [Glycine max]* | 8 | 4 | 26.04% | 21685.06 | 9.45 |
| ACU16398.1 | 40S ribosomal protein S8 | 5 | 3 | 19.37% | 21999.48 | 9.8 |
| ACU16241.1 | PREDICTED: 60S ribosomal protein L13a-4-like [Glycine max]* | 4 | 2 | 14.79% | 15757.43 | 10.37 |
| ACU16074.1 | PREDICTED: 40S ribosomal protein S17-4 [Glycine max]* | 35 | 9 | 47.03% | 26282.71 | 9.73 |
| ACU15827.1 | 40S ribosomal protein S7, putative [Ricinus communis] * | 4 | 3 | 19.28% | 28417.16 | 10.77 |
| ACU15099.1 | PREDICTED: 60S ribosomal protein L18-3-like [Glycine max]* | 6 | 4 | 20.31% | 28195.45 | 10.99 |
| ACU15002.1 | ribosomal protein L17 [Castanea sativa] * | 2 | 2 | 12.73% | 29016.4 | 10.07 |
| ACU14901.1 | PREDICTED: 60S ribosomal protein L35-like [Glycine max]* | 1 | 1 | 10.57% | 14348.37 | 10.83 |
| ACU14840.1 | Putative uncharacterized protein | 7 | 5 | 22.77% | 26268.56 | 9.81 |
| ACU14744.1 | 60S ribosomal protein L21 [Medicago truncatula] | 5 | 4 | 18.29% | 18662.56 | 10.5 |
| ACU14587.1 | PREDICTED: 60S ribosomal protein L7-4-like [Glycine max]* | 3 | 1 | 15.12% | 9642.17 | 9.08 |
| ACU14554.1 | 60S ribosomal protein L32, putative [Ricinus communis]* | 1 | 1 | 3.65% | 29850.51 | 10.8 |
| ACU14471.1 | PREDICTED: 60S ribosomal protein L13a-4-like [Glycine max]* | 6 | 4 | 22.22% | 23054.02 | 9.92 |
| ACU14439.1 | Ribosomal protein S27 | 3 | 1 | 16.07% | 12794.96 | 10.61 |
| ACU14284.1 | 60S ribosomal protein L7, putative [Ricinus communis]* | 1 | 1 | 6.00% | 16888.61 | 10.7 |
| ACU14254.1 | 60S ribosomal protein L13 | 4 | 2 | 11.72% | 29129.54 | 10.26 |
| ACU14234.1 | PREDICTED: 60S ribosomal protein L34-like [Glycine max]* | 2 | 1 | 6.16% | 16778.45 | 11.02 |
| ACU14196.1 | PREDICTED: 60S ribosomal protein L28-2 [Glycine max]* | 8 | 5 | 19.68% | 33906.54 | 5.1 |
| ACU14165.1 | PREDICTED: elongation factor 1-beta 1-like [Glycine max]* | 4 | 3 | 19.20% | 24327.36 | 4.56 |
| ACU14150.1 | PREDICTED: probable 60S ribosomal protein L14-like [Glycine max]* | 4 | 3 | 19.34% | 20713.1 | 9.97 |
| ACU14050.1 | PREDICTED: 60S ribosomal protein L9-like isoform 1 [Glycine max]* | 7 | 4 | 18.18% | 28385.21 | 9.82 |
| ACU14014.1 | PREDICTED: elongation factor 1-delta-like [Glycine max]* | 6 | 3 | 13.48% | 25087.32 | 4.46 |
| ACU13959.1 | PREDICTED: 40S ribosomal protein S26-1-like [Glycine max]* | 1 | 1 | 7.81% | 21685.06 | 9.45 |
| ACU13949.1 | eukaryotic translation initiation factor 5A3 [Glycine max]* | 8 | 4 | 25.00% | 17476.66 | 5.66 |
| ACU13905.1 | 60S ribosomal protein L37a, putative [Ricinus communis]* | 17 | 7 | 26.52% | 29897.87 | 10.22 |
| ACU13660.1 | PREDICTED: 60S ribosomal protein L18-3-like [Glycine max]* | 3 | 2 | 14.44% | 20980.76 | 11.18 |
| ACU13586.1 | PREDICTED: 40S ribosomal protein S15-like isoform 1 [Glycine max]* | 5 | 2 | 18.18% | 15889.35 | 10.18 |
| ACU13479.1 | PREDICTED: 60S ribosomal protein L35-like [Glycine max] * | 5 | 3 | 12.22% | 34099.58 | 5.11 |
| ACU13431.1 | Putative ribosomal protein S15 | 2 | 2 | 8.80% | 24512.99 | 9.85 |
| ACU13335.1 | 60S ribosomal protein L31 [Medicago truncatula]* | 3 | 2 | 12.16% | 25064.28 | 10.36 |
| ACU13169.1 | PREDICTED: 40S ribosomal protein S7 [Glycine max]* | 13 | 6 | 26.90% | 23048.63 | 10.37 |
| AAL82617.1 | elongation factor 1-gamma | 3 | 2 | 7.38% | 47710.76 | 6.3 |
| AAL18814.1 | translationally controlled tumor-like protein | 9 | 2 | 16.67% | 18995.64 | 4.57 |
| AAB63814.1 | acidic ribosomal protein P0 | 13 | 6 | 34.17% | 34189.26 | 5.02 |
| ACU14830.1 | 60S ribosomal protein L22-like protein [Medicago truncatula]* | 3 | 2 | 21.01% | 13521.49 | 9.57 |
| ACU17802.1 | PREDICTED: translocon-associated protein subunit alpha-like [Glycine max]* | 2 | 1 | 5.04% | 27778.56 | 4.84 |
| ACU14561.1 | Translocon-associated protein subunit beta [Medicago truncatula]* | 2 | 1 | 8.25% | 20949.34 | 9.36 |
| **Targeting (1)** | | | | | | |
| ACU14908.1 | microsomal signal peptidase 23 kD subunit, putative [Ricinus communis]* | 1 | 1 | 9.58% | 19239.9 | 7.87 |
| **Post-translational modification (12)** | | | | | | |
| ACU17640.1 | Acid phosphatase 1 precursor, putative [Ricinus communis]* | 1 | 1 | 3.16% | 32935.88 | 8.46 |
| ACU22724.1 | type 1 protein phosphatase-1 [Vicia faba]* | 1 | 1 | 6.33% | 35947.49 | 5.18 |
| AAD51626.1 | Poly [ADP-ribose] polymerase 3 | 42 | 21 | 38.65% | 91689.36 | 5.41 |
| ACU13350.1 | protein phosphatase 2c, putative [Ricinus communis]* | 1 | 1 | 8.54% | 18340.8 | 4.99 |
| ACU20067.1 | Peptide methionine sulfoxide reductase [Medicago truncatula]* | 1 | 1 | 5.42% | 22761.32 | 5.84 |
| ACJ37400.1 | serine/threonine kinase-like protein | 1 | 1 | 2.20% | 66249.42 | 5.87 |
| ACM89577.1 | leucine-rich repeat family protein / protein kinase family protein | 1 | 1 | 2.46% | 76741.03 | 5.64 |
| ACM89523.1 | protein kinase family protein | 1 | 1 | 2.63% | 79274.13 | 6.22 |
| ACY36006.1 | EDR1 | 1 | 1 | 1.49% | 97404.4 | 6.18 |
| CBC97662.1 | putative calcium dependent protein kinase [Trifolium pratense]* | 1 | 1 | 3.40% | 58721.53 | 5.82 |
| ACU16907.1 | Putative uncharacterized protein | 2 | 1 | 5.71% | 19745.54 | 7.66 |
| ACU18093.1 | ubiquitin-protein ligase, putative [Ricinus communis]* | 1 | 1 | 3.49% | 27012.59 | 8.69 |
| **Degradation (56)** | | | | | | |
| ACU24389.1 | PREDICTED: 26S proteasome non-ATPase regulatory subunit 11-like isoform 1 [Glycine max]* | 6 | 3 | 9.48% | 46835.43 | 5.78 |
| P01070.2 | Trypsin inhibitor A; | 5 | 3 | 12.04% | 24005.33 | 4.99 |
| CBD35142.1 | SKP1-like a [Medicago truncatula]* | 1 | 1 | 12.26% | 17466.69 | 4.56 |
| CBD35029.1 | Ubiquitin carboxyl-terminal hydrolase, putative [Ricinus communis]* | 1 | 1 | 0.65% | 104403.42 | 5.71 |
| CBC72519.1 | Xaa-Pro aminopeptidase 2 [Glycine max]* | 1 | 1 | 3.87% | 49106.24 | 5.39 |
| CBC59479.1 | Proteasome component (PCI) domain protein [Arabidopsis thaliana]* | 1 | 1 | 5.12% | 46157.35 | 4.91 |
| CBC57853.1 | aspartyl protease family protein [Arabidopsis thaliana]* | 2 | 1 | 2.66% | 51954.35 | 8.11 |
| CBC57392.1 | Aspartic proteinase nepenthesin-2 precursor, putative [Ricinus communis]* | 1 | 1 | 4.24% | 50141.24 | 8.98 |
| CBC56170.1 | cullin-like protein1 [Pisum sativum]* | 1 | 1 | 0.94% | 86703.77 | 6.69 |
| CBC52029.1 | ATSUG1 [Arabidopsis lyrata subsp. lyrata]* | 3 | 2 | 5.98% | 47088.69 | 8.85 |
| CBC52011.1 | 26S proteasome subunit 7-like protein [Lactuca sativa]* | 4 | 3 | 10.80% | 47711.02 | 6.14 |
| CAV33729.1 | proteasome-like protein alpha subunit-like [Solanum tuberosum]* | 2 | 2 | 11.06% | 25578.13 | 5.51 |
| ACU24043.1 | ATP-dependent Clp protease proteolytic subunit | 1 | 1 | 5.22% | 25624.09 | 9.48 |
| ACU23791.1 | Proteasome subunit beta type | 8 | 4 | 22.43% | 29230.03 | 5.77 |
| ACU23360.1 | Cysteine proteinase [Medicago truncatula] * | 2 | 2 | 8.56% | 40197.43 | 5.65 |
| ACU23300.1 | Putative uncharacterized protein | 2 | 1 | 7.65% | 19078.5 | 4.46 |
| ACU22821.1 | 26S proteasome non-ATPase regulatory subunit [Cucumis melo subsp. melo]* | 3 | 3 | 12.59% | 42919.91 | 4.62 |
| ACU21161.1 | Proteasome subunit alpha type | 5 | 3 | 15.61% | 25980.31 | 4.7 |
| ACU20945.1 | 26S proteasome non-atpase regulatory subunit, putative [Ricinus communis]* | 2 | 1 | 7.56% | 13756.23 | 9.16 |
| ACU20849.1 | Proteasome subunit alpha type | 5 | 2 | 10.04% | 27265.13 | 5.6 |
| ACU20595.1 | Proteasome subunit beta type | 10 | 3 | 31.88% | 22601.57 | 6.88 |
| ACU20450.1 | PREDICTED: serpin-ZX-like [Glycine max]* | 2 | 1 | 9.02% | 14285.42 | 6.71 |
| ACU20389.1 | PREDICTED: ubiquitin thioesterase otubain-like [Glycine max]* | 1 | 1 | 3.32% | 33803.13 | 4.79 |
| ACU19914.1 | 26S proteasome subunit RPN5b [Elaeis guineensis]* | 2 | 1 | 3.63% | 44551.4 | 5.39 |
| ACU19677.1 | PREDICTED: proliferation-associated protein 2G4-like [Glycine max]* | 6 | 3 | 10.15% | 43494.85 | 6.26 |
| ACU19278.1 | Proteasome subunit beta type | 2 | 2 | 7.01% | 29301.57 | 6.2 |
| ACU19069.1 | PREDICTED: kunitz-type trypsin inhibitor KTI1-like [Glycine max]* | 7 | 4 | 16.39% | 25946.94 | 8.57 |
| ACU19069.1 | PREDICTED: kunitz-type trypsin inhibitor KTI1-like [Glycine max]* | 48 | 8 | 32.87% | 24067.34 | 4.9 |
| ACU18997.1 | Proteasome subunit alpha type | 14 | 5 | 30.89% | 27392.09 | 5.83 |
| ACU18966.1 | Proteasome subunit alpha type | 4 | 2 | 15.60% | 27180.91 | 7.6 |
| ACU17665.1 | Putative uncharacterized protein | 6 | 3 | 19.85% | 30806.32 | 5.04 |
| ACU14208.1 | Proteasome subunit beta type [Medicago truncatula] * | 5 | 2 | 9.80% | 22582.9 | 5.86 |
| ACU14087.1 | Proteasome subunit beta type | 3 | 2 | 15.69% | 22783.31 | 5.47 |
| ACU13737.1 | trypsin inhibitor A precursor [Glycine max]* | 40 | 9 | 60.10% | 22446.82 | 4.85 |
| ACU13677.1 | Proteasome subunit beta type | 4 | 3 | 20.17% | 26356.18 | 6.61 |
| ACU13218.1 | Proteasome subunit beta type | 4 | 2 | 10.30% | 25128.22 | 5.3 |
| ACU13164.1 | Proteasome subunit beta type | 4 | 2 | 15.69% | 22816.36 | 5.32 |
| ACS49839.1 | trypsin inhibitor 26 kDa isoform | 5 | 4 | 20.17% | 25946.94 | 8.57 |
| ACS49839.1 | trypsin inhibitor 26 kDa isoform | 13 | 2 | 13.79% | 22986.45 | 7.6 |
| AAF87095.1 | trypsin inhibitor | 6 | 1 | 11.31% | 17976.47 | 6.12 |
| AAF70292.1 | 20S proteasome subunit | 6 | 2 | 15.60% | 27503.2 | 6.61 |
| AAF65315.1 | kunitz trypsin inhibitor 3 | 3 | 1 | 5.91% | 22986.45 | 7.6 |
| AAC28135.1 | proteasome IOTA subunit | 5 | 2 | 13.25% | 27101.68 | 6.98 |
| AAB23483.1 | Kunitz trypsin inhibitor KTi2 | 2 | 2 | 12.25% | 22800.29 | 6.14 |
| AAB23482.1 | Kunitz trypsin inhibitor KTi1 | 6 | 4 | 19.70% | 22545.96 | 4.97 |
| AAB09252.1 | 34 kDa maturing seed vacuolar thiol protease precursor | 32 | 5 | 16.09% | 42775.78 | 5.65 |
| AAA33954.1 | protease inhibitor IV | 1 | 1 | 15.66% | 9467.76 | 4.93 |
| 1AVU-A | Trypsin Inhibitor A | 128 | 14 | 70.17% | 20094.71 | 4.61 |
| CBC57789.1 | Peptidase A1, pepsin [Medicago truncatula]* | 1 | 1 | 2.56% | 55509.7 | 6.28 |
| ACU19150.1 | Intracellular protease [Medicago truncatula]* | 1 | 1 | 4.28% | 39766.52 | 5.25 |
| CAY04641.1 | PREDICTED: presequence protease 2, chloroplastic/mitochondrial-like [Glycine max]* | 1 | 1 | 1.48% | 121365.85 | 5.78 |
| ACU19503.1 | PREDICTED: gamma-interferon-inducible lysosomal thiol reductase-like [Glycine max]* | 6 | 2 | 12.86% | 27799.81 | 5.23 |
| ACU20290.1 | gamma-glutamyl hydrolase precursor [Glycine max]* | 8 | 3 | 20.33% | 26922.6 | 5.43 |
| ACU20811.1 | N-carbamoyl-L-amino acid hydrolase [Medicago truncatula]* | 3 | 1 | 3.23% | 49774.73 | 5.43 |
| CBC52027.1 | PREDICTED: 26S proteasome regulatory subunit 4 homolog A-like [glycine max]* | 2 | 2 | 7.90% | 49343.93 | 5.91 |
| ACU17665.1 | PREDICTED: 26S proteasome non-ATPase regulatory subunit RPN12A-like [Glycine max]* | 6 | 3 | 19.85% | 30806.32 | 5.04 |
| **Protein folding (15)** | | | | | | |
| AAA80588.1 | calnexin | 3 | 2 | 4.40% | 62080.79 | 4.79 |
| CBA13551.1 | cytosolic chaperonin, delta subunit | 4 | 4 | 17.07% | 57655.97 | 6.85 |
| ACU24486.1 | Calreticulin-1[Nicotiana tabacum]* | 8 | 3 | 12.86% | 48173.93 | 4.44 |
| CBC46633.1 | Chaperone DnaK [Medicago truncatula]* | 2 | 2 | 4.14% | 72554.07 | 5.82 |
| CBC96536.1 | calnexin, putative [Ricinus communis]* | 1 | 1 | 3.14% | 61661.37 | 4.8 |
| CBA13552.1 | cytosolic chaperonin, delta subunit | 1 | 1 | 2.25% | 57567.95 | 7.16 |
| ACU24395.1 | PREDICTED: 20 kDa chaperonin, chloroplastic-like [Glycine max]* | 1 | 1 | 4.74% | 26669.35 | 7.79 |
| ACU17027.1 | PREDICTED: probable prefoldin subunit 3 [Vitis vinifera]* | 1 | 1 | 7.18% | 21411.19 | 4.7 |
| CBC52399.1 | cyclophilin [Citrus sinensis]* | 16 | 5 | 37.21% | 18224.63 | 8.69 |
| CBC52455.1 | cyclophilin [Camellia oleifera]* | 3 | 1 | 10.78% | 21944.1 | 8.89 |
| CBC52401.1 | cyclophilin [Camellia oleifera]* | 2 | 1 | 5.88% | 21954.18 | 9.2 |
| CBC55944.1 | ARG1 protein [Arabidopsis thaliana]* | 1 | 1 | 3.90% | 45329.92 | 6.17 |
| CBC97838.1 | rubisco subunit binding-protein beta subunit, rubb, putative [Ricinus communis]* | 14 | 8 | 19.76% | 62840.86 | 5.85 |
| CBC97841.1 | putative rubisco subunit binding-protein alpha subunit [Glycine max]* | 15 | 7 | 16.95% | 61732.53 | 5.29 |
| CBC97836.1 | chaperonin-60kD, ch60, putative [Ricinus communis]* | 1 | 1 | 4.33% | 61423.53 | 5.74 |
| **Cell (48)** | | | | | | |
| ACU13224.1 | PREDICTED: actin-depolymerizing factor 2-like [Glycine max]* | 4 | 2 | 20.14% | 15978.23 | 6.15 |
| P69325.1 | Ubiquitin | 5 | 4 | 61.84% | 8524.74 | 6.56 |
| CBD32756.1 | cell division cycle protein 48 homolog [Glycine max]* | 16 | 7 | 12.52% | 89830.12 | 5.16 |
| CBD31740.1 | NAP-related protein [Medicago sativa subsp. x varia]* | 1 | 1 | 4.21% | 30090.62 | 4.16 |
| CBD12283.1 | PREDICTED: reticulon-like protein B2-like isoform 1 [Glycine max]* | 2 | 1 | 3.15% | 28567.17 | 8.39 |
| CBD12283.1 | PREDICTED: reticulon-like protein B2-like isoform 1 [Glycine max]* | 1 | 1 | 3.15% | 28567.17 | 8.39 |
| CBD10580.1 | exocyst subunit exo70 family protein B1 [Arabidopsis thaliana]* | 1 | 1 | 1.86% | 72966 | 5.24 |
| CBD05475.1 | coatomer delta subunit, putative [Ricinus communis]* | 4 | 3 | 23.83% | 22055.47 | 6.43 |
| CBD05161.1 | histone H4 [Zea mays]* | 1 | 1 | 9.71% | 11395.3 | 11.48 |
| CBC77749.1 | harpin binding protein 1 [Glycine max]* | 2 | 1 | 4.53% | 28431.59 | 7.88 |
| CBC51697.1 | cell division control protein [Paracoccidioides brasiliensis Pb18]* | 2 | 1 | 2.55% | 90380.63 | 4.95 |
| ACU24198.1 | Stomatin-like protein [Medicago truncatula]* | 3 | 2 | 8.91% | 44074.45 | 9.21 |
| ACU23590.1 | derlin-1 [Arabidopsis thaliana]* | 1 | 1 | 6.40% | 19381.16 | 10.27 |
| ACU23005.1 | Mo25-like protein [Arabidopsis thaliana] * | 1 | 1 | 2.65% | 39428 | 5.45 |
| ACU22968.1 | actin isoform PEAc14-1 [Pisum sativum]* | 8 | 5 | 23.08% | 41834.84 | 5.23 |
| ACU21032.1 | epsilon2-COP [Glycine max]* | 5 | 3 | 18.28% | 32278.61 | 5.3 |
| ACU19580.1 | PREDICTED: tubulin alpha-4 chain-like [Glycine max]* | 7 | 4 | 11.14% | 50431.73 | 4.71 |
| ACU19513.1 | PREDICTED: hypothetical protein [Vitis vinifera]* | 1 | 1 | 3.96% | 44600.96 | 7.6 |
| ACU19354.1 | similar to plectin-related [Vitis vinifera]* | 1 | 1 | 1.42% | 55186.98 | 6.89 |
| ACU19151.1 | Mitochondrial acidic protein mam33 [Medicago truncatula]* | 1 | 1 | 5.68% | 26688.4 | 6.45 |
| ACU19016.1 | vesicle-associated membrane family protein [Arabidopsis thaliana]* | 1 | 1 | 6.42% | 30028.28 | 8.55 |
| ACU18050.1 | tubulin A [Glycine max]* | 8 | 5 | 17.11% | 49606.23 | 5.03 |
| ACU17933.1 | actin [Vigna radiata]* | 15 | 7 | 32.89% | 41696.81 | 5.31 |
| ACU17772.1 | GTP-binding protein SAR1A [Medicago truncatula]* | 6 | 3 | 24.87% | 22084.5 | 5.95 |
| ACU16645.1 | Peroxisomal biogenesis factor 11 family protein | 2 | 1 | 7.23% | 25902.25 | 9.78 |
| ACU15951.1 | PREDICTED: GTP-binding protein SAR1A-like [Glycine max]* | 7 | 3 | 23.83% | 22192.64 | 6.91 |
| ACU14984.1 | PREDICTED: GTP-binding protein SAR1A [Glycine max]* | 10 | 4 | 32.64% | 22132.52 | 5.97 |
| ACU14661.1 | PREDICTED: GTP-binding protein SAR1A-like [Glycine max]* | 12 | 5 | 37.31% | 22119.5 | 6.52 |
| ACU14486.1 | oleosin 2 [Arachis hypogaea]* | 16 | 5 | 19.73% | 23392.66 | 8.89 |
| ACU14320.1 | PREDICTED: actin-depolymerizing factor 2-like [Glycine max]* | 1 | 1 | 8.63% | 15995.27 | 5.91 |
| ACU14142.1 | tubulin A [Glycine max]* | 8 | 4 | 23.50% | 20126.81 | 9.69 |
| ACU13416.1 | PREDICTED: GTP-binding protein SAR1A-like [Glycine max]* | 3 | 2 | 5.28% | 58406.3 | 5.61 |
| ACU13271.1 | Alba DNA/RNA-binding protein [Arabidopsis thaliana]* | 1 | 1 | 19.44% | 15893.03 | 5.58 |
| ACU13135.1 | 16.5 kDa oleosin [Glycine max]* | 10 | 4 | 15.49% | 23501.64 | 8.02 |
| AAX86048.1 | tubulin B4 | 3 | 3 | 9.58% | 49471.93 | 4.96 |
| AAC49294.1 | clathrin heavy chain | 3 | 3 | 3.53% | 193355.53 | 5.37 |
| AAC05272.1 | actin 4 | 1 | 1 | 1.86% | 41314.33 | 5.6 |
| AAB71227.1 | Ca+2-binding EF hand protein | 4 | 2 | 12.13% | 26979.66 | 5.98 |
| AAB40081.1 | actin | 11 | 4 | 22.02% | 37249.75 | 5.82 |
| AAB40079.1 | actin | 9 | 5 | 28.27% | 37165.54 | 5.47 |
| AAB40078.1 | actin | 8 | 4 | 22.92% | 37126.51 | 5.28 |
| AAA17855.1 | p24 oleosin isoform B | 5 | 3 | 27.88% | 17416.82 | 7.75 |
| AAA17854.1 | p24 oleosin isoform A | 7 | 2 | 23.03% | 17416.82 | 7.75 |
| ACU20139.1 | PREDICTED: protein SEC13 homolog [Glycine max]* | 3 | 1 | 3.99% | 32531.3 | 5.46 |
| ACU13783.1 | Mitochondria fission 1 protein | 1 | 1 | 11.76% | 18690.56 | 6.96 |
| CBD35351.1 | PREDICTED: 65-kDa microtubule-associated protein 3-like [Glycine max]* | 2 | 1 | 2.08% | 65736.64 | 5.78 |
| ACU21146.1 | prohibitin, putative [Ricinus communis] * | 4 | 1 | 6.57% | 31831.76 | 9.72 |
| ACU18985.1 | PREDICTED: prohibitin-2-like [Glycine max]* | 1 | 1 | 6.57% | 31818.49 | 9.42 |
| **Development (22)** | | | | | | |
| ACT80135.1 | PREDICTED: interferon-related developmental regulator 1-like [Glycine max]* | 2 | 1 | 4.68% | 49427.03 | 6.74 |
| CBC52008.1 | regulatory particle triple-A ATPase 5A [Arabidopsis thaliana]* | 4 | 3 | 10.40% | 47455.18 | 4.98 |
| ACU16281.1 | seed maturation protein PM34 [Glycine max]* | 1 | 1 | 4.65% | 24274.98 | 8.35 |
| ACU16211.1 | 51 kDa seed maturation protein [Glycine max]* | 1 | 1 | 10.00% | 16250 | 10.79 |
| ACU13481.1 | PREDICTED: developmentally regulated G-protein 2 [Glycine max]* | 1 | 1 | 6.27% | 30077.77 | 8.87 |
| ACU13211.1 | translationally-controlled tumor protein homolog [Glycine max]* | 6 | 2 | 20.36% | 18863.47 | 4.63 |
| AAG37440.1 | seed maturation protein LEA 4 | 4 | 1 | 6.94% | 17606.09 | 9.58 |
| AAF89645.1 | seed maturation protein PM34 | 77 | 10 | 50.17% | 31767.03 | 6.6 |
| AAF21311.1 | seed maturation protein PM25 | 46 | 12 | 81.42% | 25728.65 | 4.99 |
| AAF21310.1 | seed maturation protein PM24 | 12 | 5 | 35.14% | 26840.63 | 5.14 |
| AAD51627.1 | seed maturation protein PM39 | 10 | 3 | 9.52% | 46692.85 | 5.69 |
| AAD51624.1 | seed maturation protein PM36 | 30 | 7 | 37.12% | 25987.72 | 5.26 |
| AAD49719.1 | maturation protein pPM32 | 3 | 1 | 7.51% | 18882.3 | 5.49 |
| AAD30865.1 | seed maturation protein PM31 | 42 | 6 | 47.06% | 17746.85 | 6.1 |
| AAD30864.1 | seed maturation protein PM30 | 2 | 1 | 9.29% | 15097.25 | 8.95 |
| AAD01540.1 | 24 kDa seed maturation protein | 2 | 2 | 12.26% | 24174.44 | 9.19 |
| AAD01431.1 | 35 kDa seed maturation protein | 4 | 2 | 6.65% | 35342.03 | 5.96 |
| AAC61783.1 | LEA protein | 79 | 18 | 41.52% | 67877.91 | 6.1 |
| AAA91965.1 | 51 kDa seed maturation protein | 25 | 16 | 38.27% | 50982.09 | 6.65 |
| AAA33985.1 | late embryongenesis abundant protein | 14 | 6 | 18.57% | 50643.63 | 6.33 |
| ACU23559.1 | 51 kDa seed maturation protein precursor  [Glycine max]* | 17 | 12 | 26.42% | 49335.41 | 7.08 |
| ACU13245.1 | Embryo-specific protein [Medicago truncatula]* | 2 | 1 | 9.09% | 20732.4 | 4.57 |
| **Stress (55)** | | | | | | |
| ACT65757.1 | stachyose synthase | 2 | 1 | 5.26% | 48546.46 | 5.38 |
| ACU23585.1 | Fe-S cluster assembly protein DRE2 homolog | 1 | 1 | 8.85% | 27938.24 | 4.85 |
| Q43468.1 | Heat shock protein STI; | 1 | 1 | 2.64% | 63585.27 | 5.81 |
| P26413.1 | Heat shock 70 kDa protein | 3 | 2 | 24.39% | 17946.47 | 5.6 |
| P05478.1 | 18.5 kDa class I heat shock protein | 28 | 14 | 29.10% | 73893.88 | 5.08 |
| CBD35522.1 | BCL-2-associated athanogene 7 [Arabidopsis thaliana]* | 1 | 1 | 1.99% | 46353.02 | 9.42 |
| CBD34754.1 | heat shock protein 70 [Gossypium hirsutum]* | 16 | 8 | 16.90% | 70879.49 | 5.37 |
| CBD34753.1 | heat shock protein, putative [Ricinus communis]* | 8 | 4 | 8.57% | 80157.17 | 4.98 |
| CBD08931.1 | Haem peroxidase, plant/fungal/bacterial [Medicago truncatula]* | 1 | 1 | 5.10% | 34622.63 | 9.11 |
| CBC46637.1 | heat shock protein, putative [Ricinus communis]* | 1 | 1 | 8.47% | 19558.66 | 5.9 |
| CBC46634.1 | heat shock protein 70 [Cucumis sativus]* | 7 | 4 | 29.81% | 18502.92 | 5.82 |
| CBC46629.1 | heat shock protein 70 [Gossypium hirsutum]* | 2 | 1 | 11.59% | 17735.19 | 5.68 |
| CBC46619.1 | heat shock protein 70 [Cucumis sativus]* | 1 | 1 | 7.56% | 26011.09 | 5.39 |
| CBC46617.1 | heat shock protein 70 [Gossypium hirsutum]* | 10 | 5 | 36.42% | 18022.73 | 6.72 |
| CAE47768.1 | dehydrin | 9 | 4 | 32.64% | 25368.54 | 6.19 |
| ACU24346.1 | Putative uncharacterized protein | 1 | 1 | 2.86% | 47114.07 | 6.65 |
| ACU24076.1 | Universal stress protein A-like protein [Medicago truncatula]* | 26 | 12 | 24.92% | 71499.86 | 5.05 |
| ACU20991.1 | Putative uncharacterized protein | 2 | 1 | 9.43% | 17000.6 | 7.72 |
| ACU20570.1 | BCL-2-associated athanogene 6 [Arabidopsis thaliana]* | 1 | 1 | 9.09% | 19951.39 | 4.85 |
| ACU20291.1 | pyrimidine 2 [Arabidopsis thaliana]* | 1 | 1 | 2.09% | 57677.16 | 5.91 |
| ACU17048.1 | 17.3 kDa class I heat shock protein [Glycine max]* | 4 | 2 | 9.80% | 17436.78 | 5.52 |
| ACU16890.1 | stress-induced protein SAM22 [Glycine max]* | 6 | 3 | 24.05% | 16760.76 | 4.73 |
| ACU15913.1 | PREDICTED: 17.5 kDa class I heat shock protein-like [Glycine max]* | 3 | 3 | 20.26% | 17513.82 | 5.72 |
| ACU15563.1 | PREDICTED: 18.2 kDa class I heat shock protein [Glycine max]* | 12 | 5 | 53.50% | 17740.02 | 6.33 |
| ACU14933.1 | Disease resistance response protein [Medicago truncatula]* | 2 | 1 | 6.42% | 20536.03 | 5.18 |
| ACU14709.1 | universal stress protein 1 [Gossypium arboreum] * | 13 | 8 | 17.79% | 73580.44 | 5.1 |
| ACU13778.1 | disease resistance response protein 1 [Glycine max]* | 1 | 1 | 4.19% | 21230.43 | 5.9 |
| ACU13396.1 | universal stress protein 1 [Gossypium arboreum]* | 25 | 11 | 20.99% | 73568.34 | 5.08 |
| ACU13353.1 | Universal stress protein A-like protein [Medicago truncatula]* | 22 | 9 | 16.95% | 71232.66 | 5.09 |
| ACM89635.1 | disease-resistance protein | 2 | 1 | 3.91% | 64772.86 | 6.23 |
| ACI31552.1 | heat shock protein 90-1 | 5 | 3 | 5.70% | 80465.52 | 4.94 |
| ACI31551.1 | heat shock protein 90-2 | 24 | 11 | 25.27% | 71081.33 | 5.11 |
| ACC60273.1 | aluminum-activated malate transporter | 1 | 1 | 2.26% | 53599.08 | 7.23 |
| AAR19096.1 | NBS-LRR type disease resistance protein RPG1-B | 1 | 1 | 1.31% | 137976.66 | 5.83 |
| AAQ02338.1 | lea protein | 24 | 9 | 71.68% | 23787.61 | 5.97 |
| AAK21920.1 | BiP-isoform D | 2 | 1 | 3.64% | 54263.23 | 6.95 |
| AAG48132.1 | putative resistance protein | 2 | 1 | 1.37% | 124977.84 | 6.16 |
| AAD25354.1 | seed maturation protein PM22 | 7 | 5 | 34.87% | 16688.32 | 5.16 |
| AAB86942.1 | endoplasmic reticulum HSC70-cognate binding protein precursor | 25 | 12 | 24.55% | 73639.53 | 5.15 |
| AAB19129.1 | seed coat peroxidase isozyme | 1 | 1 | 6.01% | 30600.19 | 5.07 |
| AAA66338.1 | heat shock protein | 5 | 3 | 5.49% | 101328.84 | 5.85 |
| AAA33991.1 | maturation-associated protein | 25 | 9 | 71.68% | 23717.51 | 6.07 |
| AAA33973.1 | Gmhsp26-A | 26 | 12 | 24.10% | 73633.67 | 5.11 |
| AAA18834.1 | dehydrin-like protein | 8 | 4 | 35.80% | 25658.76 | 6.02 |
| AAK21920.1 | BiP-isoform D | 3 | 2 | 6.46% | 54263.23 | 6.95 |
| AAA81954.1 | BiP isoform B | 25 | 12 | 24.92% | 73491.43 | 5.11 |
| AAA81954.1 | BiP isoform B | 1 | 1 | 1.86% | 41314.33 | 5.6 |
| ACU17980.1 | PREDICTED: (+)-neomenthol dehydrogenase-like [Glycine max]* | 5 | 2 | 10.70% | 32475.07 | 5.39 |
| ACU24382.1 | pathogenesis-related protein [Arabidopsis thaliana]* | 1 | 1 | 8.79% | 20689.9 | 7.88 |
| CBC62813.1 | PREDICTED: NADP-dependent alkenal double bond reductase P1-like [Glycine max] * | 10 | 4 | 20.99% | 37920.5 | 5.94 |
| ACU20720.1 | PREDICTED: heme-binding protein 2-like [Glycine max]* | 1 | 1 | 8.97% | 25931.59 | 6.83 |
| ACU23077.1 | PREDICTED: pathogen-related protein [Vitis vinifera]* | 6 | 3 | 19.75% | 27230.01 | 6.55 |
| ACU19618.1 | class III acidic endochitinase precursor [Glycine max]* | 4 | 3 | 14.12% | 36603.38 | 8.88 |
| ACU14269.1 | PRp27-like protein [Olea europaea subsp. europaea]* | 2 | 1 | 6.22% | 25237.39 | 8.72 |
| CBD10948.1 | PREDICTED: benzyl alcohol O-benzoyltransferase-like [Glycine max]* | 1 | 1 | 4.42% | 52885.22 | 6.23 |
| **Storage protein (33)** | | | | | | |
| BAC78524.1 | prepro beta-conglycinin alpha prime subunit | 179 | 23 | 32.83% | 69887.4 | 5.43 |
| PRF:224389 | glycinin A5 | 4 | 2 | 10.67% | 38319.84 | 5.82 |
| P13917.2 | Basic 7S globulin | 628 | 30 | 73.56% | 47907.86 | 5.67 |
| P13916.2 | Beta-conglycinin, alpha chain | 707 | 42 | 60.50% | 70306.64 | 5.12 |
| P11828.1 | Glycinin G3 | 272 | 13 | 43.60% | 54359.85 | 5.28 |
| CAA37044.1 | glycinin | 82 | 11 | 28.28% | 60038.68 | 5.65 |
| BAE46788.1 | beta-conglycinin alpha subunit | 155 | 15 | 34.88% | 70293.56 | 5.07 |
| BAD98463.1 | beta-conglycinin beta subunit | 294 | 21 | 30.66% | 72475.88 | 5.32 |
| BAD72975.1 | glycinin A5A4B3 | 570 | 16 | 36.07% | 63679.55 | 5.38 |
| BAC78524.1 | prepro beta-conglycinin alpha prime subunit | 46 | 6 | 7.57% | 96300.65 | 6.29 |
| BAB64304.1 | beta-conglycinin alpha-subunit | 201 | 13 | 26.16% | 72475.88 | 5.32 |
| BAB64303.1 | beta-conglycinin alpha prime subunit | 468 | 33 | 51.85% | 72138.32 | 5.6 |
| ACU20988.1 | Glutelin type-A [Medicago truncatula]* | 3 | 2 | 45.36% | 10678.06 | 5.86 |
| ACU18513.1 | PREDICTED: beta-conglycinin, beta chain-like [Glycine max]* | 58 | 14 | 47.86% | 48330.36 | 5.67 |
| ACT53401.1 | mutant glycinin subunit A1aB1b | 19 | 11 | 38.35% | 54390.83 | 5.46 |
| ACN11532.1 | mutant glycinin A3B4 | 439 | 16 | 37.83% | 63797.54 | 5.17 |
| ACD36978.1 | Gly m Bd 28K allergen | 879 | 34 | 51.85% | 72247.37 | 5.51 |
| ABH09130.1 | beta-conglycinin alpha'-subunit | 7 | 3 | 10.33% | 50744.95 | 5.81 |
| AAO48716.1 | sucrose-binding protein 2 | 103 | 16 | 36.61% | 55834.47 | 6.32 |
| AAO45103.1 | beta-conglycinin alpha' subunit | 700 | 37 | 54.11% | 72247.37 | 5.51 |
| AAF05723.1 | sucrose binding protein homolog S-64 | 211 | 13 | 41.58% | 54241.86 | 5.73 |
| AAD09630.1 | napin-type 2S albumin 1 precursor | 9 | 3 | 21.29% | 17834.66 | 5.99 |
| AAD00178.1 | napin-type 2S albumin 3 | 5 | 1 | 7.59% | 18459.97 | 5.2 |
| AAB23212.1 | glycinin G4 subunit | 356 | 22 | 30.50% | 72475.88 | 5.32 |
| AAB23211.1 | glycinin G3 subunit | 580 | 11 | 25.66% | 60038.68 | 5.65 |
| AAB23210.1 | glycinin G2 subunit | 412 | 18 | 52.53% | 55706.42 | 5.89 |
| AAB23209.1 | glycinin G1 subunit | 451 | 19 | 61.65% | 54390.83 | 5.46 |
| 2D5H-F | Glycinin A3B4 subunit | 15 | 7 | 22.63% | 55706.42 | 5.89 |
| 1UIJ-F | Beta-conglycinin, beta chain | 249 | 27 | 43.14% | 70293.56 | 5.07 |
| 1IPJ-C | Beta-conglycinin, beta chain | 641 | 31 | 44.30% | 72475.88 | 5.32 |
| AAF89646.1 | seed maturation protein PM40 | 1 | 1 | 3.02% | 55284.94 | 6.22 |
| Q8RVH5.1 | Basic 7S globulin2 | 5 | 3 | 8.08% | 47204.87 | 8.58 |
| P13917.2 | Basic 7S globulin | 5 | 3 | 9.37% | 46392.96 | 8.68 |
| **Not assigned(53)** | | | | | | |
| CBD29965.1 | PREDICTED: hypothetical protein [Vitis vinifera]* | 2 | 1 | 11.63% | 19315.02 | 6.16 |
| CBD21537.1 | yth domain-containing protein, putative [Ricinus communis]* | 1 | 1 | 2.42% | 63781.58 | 6.58 |
| CBD19445.1 | conserved hypothetical protein [Ricinus communis]* | 1 | 1 | 2.34% | 66152.36 | 9.07 |
| CAA80491.1 | Lea protein | 3 | 2 | 5.68% | 49399.49 | 7.08 |
| ACU24371.1 | Putative uncharacterized protein | 1 | 1 | 3.36% | 30457.89 | 5.33 |
| ACU24229.1 | Putative uncharacterized protein | 1 | 1 | 6.37% | 35355.91 | 8.43 |
| ACU24151.1 | Putative uncharacterized protein | 1 | 1 | 3.57% | 34015.75 | 6 |
| ACU23913.1 | Putative uncharacterized protein | 1 | 1 | 12.35% | 18744.65 | 9.8 |
| ACU23297.1 | Putative uncharacterized protein | 1 | 1 | 8.46% | 36239.46 | 7.05 |
| ACU23106.1 | LOB domain-containing protein, putative [Ricinus communis]* | 1 | 1 | 7.87% | 19836.6 | 8.28 |
| ACU21224.1 | PREDICTED: hypothetical protein [Vitis vinifera]* | 1 | 1 | 5.07% | 38443.38 | 6.8 |
| ACU21075.1 | Putative uncharacterized protein | 1 | 1 | 4.09% | 52299.14 | 8.22 |
| ACU20619.1 | Putative uncharacterized protein | 1 | 1 | 9.09% | 11509.33 | 5.48 |
| ACU18958.1 | conserved hypothetical protein [Ricinus communis]* | 1 | 1 | 4.55% | 40363.14 | 8.08 |
| ACU18633.1 | Putative uncharacterized protein | 1 | 1 | 6.38% | 15770.67 | 6.91 |
| ACU18556.1 | hypothetical protein LOC100306651 [Glycine max]* | 1 | 1 | 4.20% | 37095.21 | 10.4 |
| ACU18354.1 | predicted protein [Populus trichocarpa]* | 1 | 1 | 4.74% | 22823.91 | 9.57 |
| ACU17369.1 | Putative uncharacterized protein | 1 | 1 | 20.00% | 6022.01 | 10.73 |
| ACU17139.1 | Putative uncharacterized protein | 1 | 1 | 16.51% | 12316.15 | 4.7 |
| ACU16336.1 | Putative uncharacterized protein | 1 | 1 | 9.43% | 18141.83 | 4.89 |
| ACU16183.1 | Putative uncharacterized protein | 1 | 1 | 5.39% | 22938.08 | 5.45 |
| ACU16005.1 | Putative uncharacterized protein | 1 | 1 | 5.91% | 25347.74 | 5.37 |
| ACU15800.1 | Putative uncharacterized protein | 3 | 2 | 11.65% | 23316.51 | 6.79 |
| ACU15402.1 | hypothetical protein LOC100500350 [Glycine max]* | 1 | 1 | 12.00% | 11451.98 | 10.84 |
| ACU14872.1 | Putative uncharacterized protein | 2 | 1 | 6.02% | 17999.88 | 5.48 |
| ACU14793.1 | Putative uncharacterized protein | 1 | 1 | 9.55% | 17237.05 | 7.67 |
| ACU14594.1 | Putative uncharacterized protein | 1 | 1 | 6.13% | 18472.51 | 9.3 |
| ACU14484.1 | Putative uncharacterized protein | 22 | 7 | 37.61% | 27048.98 | 6.42 |
| ACU14329.1 | Putative uncharacterized protein | 1 | 1 | 9.32% | 18459.34 | 8.92 |
| ACU14147.1 | Putative uncharacterized protein | 5 | 3 | 19.53% | 24917.61 | 5.74 |
| ACU13407.1 | uncharacterized protein LOC100305625 [Glycine max]* | 1 | 1 | 3.98% | 19083.52 | 4.16 |
| ACU16926.1 | Putative uncharacterized protein | 1 | 1 | 7.02% | 25246.85 | 6.46 |
| ACU21370.1 | NAD(P)-binding Rossmann-fold-containing protein [Arabidopsis thaliana]* | 6 | 4 | 17.82% | 35670.83 | 8.91 |
| ACU23474.1 | Nucleosome-binding protein, putative [Ricinus communis]* | 1 | 1 | 4.36% | 30313.78 | 4.59 |
| ACU21485.1 | PREDICTED: obg-like ATPase 1-like [Glycine max]* | 5 | 3 | 11.93% | 44438.21 | 6.36 |
| ACU18121.1 | Putative uncharacterized protein | 1 | 1 | 6.74% | 22620.15 | 10.24 |
| ACU23232.1 | PREDICTED: transmembrane protein 111-like [Glycine max]* | 1 | 1 | 7.32% | 27745.26 | 9.24 |
| ACU14938.1 | uncharacterized protein LOC100306653 [Glycine max]* | 3 | 1 | 9.30% | 14008.08 | 5.73 |
| ACU16936.1 | Putative uncharacterized protein | 1 | 1 | 5.53% | 27656.88 | 6.39 |
| ACU19949.1 | PREDICTED: probable carboxylesterase 7-like [Glycine max]* | 1 | 1 | 4.02% | 35798.76 | 5.43 |
| ACU22898.1 | PREDICTED: probable carboxylesterase 2-like [Glycine max]* | 1 | 1 | 4.06% | 35226.18 | 5.82 |
| CBC50476.1 | PREDICTED: monoacylglycerol lipase ABHD6-like [Glycine max]* | 1 | 1 | 5.96% | 34353.02 | 8.99 |
| ACU13689.1 | Acyl-coenzyme A thioesterase [Medicago truncatula]* | 1 | 1 | 11.32% | 17273.84 | 9.25 |
| ACU18591.1 | 5-oxoprolinase, putative [Ricinus communis]* | 1 | 1 | 8.82% | 32911.29 | 5.91 |
| ACU20815.1 | PREDICTED: dihydrolipoyllysine-residue succinyltransferase component of 2-oxoglutarate dehydrogenase complex 2, mitochondrial-like [Glycine max]* | 1 | 1 | 6.15% | 19497.51 | 9.9 |
| CBG02682.1 | imidazole glycerol phosphate synthase subunit hisf, putative [Ricinus communis]* | 1 | 1 | 2.41% | 62882.08 | 5.9 |
| ACU18137.1 | Clavaminate synthase-like protein [Medicago truncatula]* | 1 | 1 | 4.95% | 36054.39 | 6.26 |
| ACU14061.1 | Adenosine kinase [Medicago truncatula]* | 1 | 1 | 7.50% | 22966.83 | 9.32 |
| ACU21550.1 | PREDICTED: phosphopantothenate--cysteine ligase 1-like [Glycine max]* | 1 | 1 | 5.62% | 35909.32 | 8.37 |
| CBC50795.1 | oxidoreductase, putative [Ricinus communis]* | 1 | 1 | 6.60% | 43453.26 | 8.46 |
| ACU16008.1 | NADPH:quinone oxidoreductase [Medicago truncatula]* | 1 | 1 | 11.62% | 21547.61 | 7.91 |
| ACU19052.1 | PREDICTED: S-formylglutathione hydrolase-like [Glycine max]* | 4 | 3 | 17.19% | 32243.61 | 6.55 |
| ACU17046.1 | hypothetical protein LOC100527853 [Glycine max]* | 1 | 1 | 8.00% | 16272.7 | 9.78 |

Note: a, peptide counting; b, unique peptide counting; c, sequence coverage; * show the Blast results of this protein from NCBI database, the query coverage percent >90%; ** means this protein have more functional categories.
